# Bioinformatics Analysis Revealing the Correlation between NF-*κ*B Signaling Pathway and Immune Infiltration in Gastric Cancer

**DOI:** 10.1155/2022/5385456

**Published:** 2022-07-28

**Authors:** Qiuxiang Chen, Xiaojing Du, Pengcheng Ruan, Yumei Ye, Jin Zheng, Sunkuan Hu

**Affiliations:** ^1^Department of Ultrasonic Imaging, The First Affiliated Hospital of Wenzhou Medical University, Wenzhou, Zhejiang 325035, China; ^2^Department of Gastroenterology, Minhang Hospital, Fudan University, 170 Xinsong Road, Shanghai 201199, China; ^3^Department of General Surgery, Fenghua People's Hospital, 36 Gongyuan Road, Ningbo, Zhejiang 315502, China; ^4^Department of Neurology, Minhang Hospital, Fudan University, 170 Xinsong Road, Shanghai 201199, China; ^5^Department of Gastroenterology, The First Affiliated Hospital of Wenzhou Medical University, Wenzhou, Zhejiang 325035, China

## Abstract

Although the emerging of immunotherapy conferred a new landscape of gastric cancer (GC) treatment, its response rate was of significant individual differences. Insight into GC immune microenviroment may contribute to breaking the dilemma. To this end, the enrichment score of NF-*κ*B signaling pathway was calculated in each GC sample from The Cancer Genome Atlas (TCGA) via ssGSEA algorithm, and its association with immune infiltration was estimated. Based on NF-*κ*B-related genes, a risk score was established and its involvement in immune infiltration, tumor mutational burden (TMB), and N6-methyladenosine (M6A) modification was analyzed in GC. The results showed that NF-*κ*B signaling pathway promoted the infiltration of immune cells in GC. In addition, GC samples were divided into low- and high-risk groups according to a seven-gene (*CARD11*, *CCL21*, *GADD45B*, *LBP*, *RELB*, *TRAF1*, and *VCAM1*) risk score. Although the high-risk group displayed high immune infiltration and high expression of M6A regulatory genes, it remains in an immunosuppressive microenviroment and whereby suffers a poorer outcome. Of note, most of hub genes were related to immune infiltration and could serve as an independent prognostic biomarker. Conclusively, our study emphasized the crucial role of NF-*κ*B signaling pathway in GC immune microenviroment and provided several candidate genes that may participate in immune infiltration.

## 1. Introduction

Although the incidence of gastric cancer (GC) decreased in the past decades, it still remains as a major tumor burden all over the world, especially in East Asian regions [1]. Development of the oncotherapy provides more options to GC patients, while patients with advanced GC frequently suffer a tragic outcome. The emerging of immune checkpoint inhibitors (ICIs) is profoundly altering the therapeutic landscape across a spectrum of cancers, including GC [2]. Programmed death-1 (PD1) inhibitor Pembrolizumab is recommended by the 2021 NCCN guideline for the posterior line treatment in GC patients who had programmed death ligand − 1 (PD − L1) combined positive score (CPS) ≥ 1, tumor mutational burden- (TMB-) high (TMB-H, ≥10 mutations per megabase), microsatellite instability-high (MSI-H), or deficient mismatch repair (dMMR) [3]. Unfortunately, even under this standard, the response rate to ICIs varies greatly among distinct individuals, due to the fiendishly complicated tumor immune microenvironment. For this reason, insight of the regulatory mechanisms of cancer immunity is of paramount importance to guiding immunotherapy.

NF-*κ*B family consists of five distinct DNA-binding proteins that form various homodimers and heterodimers and thereby drives a series of signaling pathways that could control cell differentiation, proliferation, survival, invasion, angiogenesis during tumorigenesis, and progression [4]. Convincing evidences indicated that NF-*κ*B signaling pathway acts as a driver role during carcinogenesis and progression of GC [5]. It was considered as a potential therapeutic target for GC patients. More recent progress revealed that NF-*κ*B signaling pathway was involved in cancer immune evasion [6, 7]. These studies indicated that NF-*κ*B signaling pathway functions in most of cells in tumor microenvironment, such as tumor-associated macrophages (TAMs), dendritic cells (DCs), myeloid-derived suppressor cells (MDSCs), natural killer (NK) cells, natural killer T (NKT) cells, T cells, and B cells, and regulates the expression of immune checkpoints, such as PD-L1 [4, 6, 7]. However, rare studies investigated the effect of NF-*κ*B signaling pathway on GC immunity, as well as the involving mechanisms.

Interdisciplinary collaboration has been widely applied to the prevention, diagnosis, and therapy in various diseases [8, 9]. For instance, rational mathematical modeling may help in controlling infection diseases, such as the novel corona virus (COVID-19) and hepatitis B [10–12]. The rapid progress of cancer genetics and genomics boosts the establishment of a growing body of public databases, such as The Cancer Genome Atlas (TCGA) and Gene Expression Omnibus (GEO) [13]. These databases greatly accelerated the application of bioinformatics to the field of cancers. Here, the single-sample gene set enrichment analysis (ssGSEA) was employed to calculate the enrichment score of NF-*κ*B signaling pathway in each GC sample from TCGA, and its correlation with immune infiltration was analyzed in GC. Based on NF-*κ*B-related genes, a seven-gene risk score was further established by using least absolute shrinkage and selection operator (LASSO). Then, the immune infiltration and TMB as well as N6-methyladenosine (M6A) modification were evaluated in different risk score groups. Finally, the association was investigated between the seven genes and prognosis as well as immune infiltration in GC.

## 2. Materials and Methods

**Figure 1:**
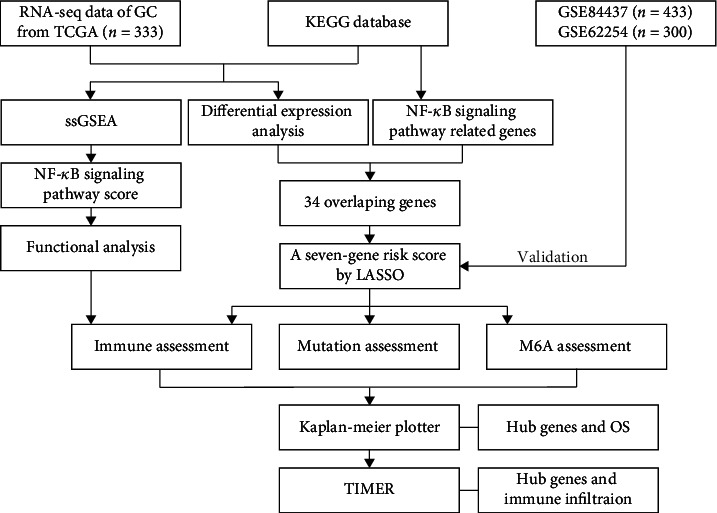
The overview of the analytic procedure in this study.

The overall study processes are shown in Figure 1.

### 2.1. Data Download

GC RNA sequencing (RNA-seq) data (32 normal and 375 tumor) and related clinical data were downloaded from TCGA database, among which 42 samples were excluded for their follow-up time or survival time less than 30 days. GSE62254 (*n* = 300) and GSE84437 (*n* = 433) with survival data were downloaded from GEO database. The clinical characteristics of GC samples were displayed in Table S1.

### 2.2. Differentially Expressed Genes and NF-*κ*B-Related Genes

Differentially expressed genes (DEGs) were identified by using “limma” package of R (version 4.1.0) [14]. The cutoff values were set as follows: ∣log_2_FC (fold change) | >1 and adj.*p*.val (adjusted *p* value) < 0.05. NF-*κ*B related genes (*n* = 104) were obtained from Kyoto Encyclopedia of Genes and Genomes (KEGG) database (Figure S1) [15].

### 2.3. Gene Set Enrichment Analysis

Gene set enrichment analysis (GSEA) was used to reveal the underlying pathways [16]. Based on the median enrichment score, GC samples were classified into low (C1) and high (C2) NF-*κ*B signaling pathway groups. The “clusterProfiler” package of R was applied to conduct the biological process (BP) analysis [17]. ∣NES | >1 and adj.*p*.val < 0.05 were considered as a significant result.

### 2.4. Gene Set Variation Analysis

Gene set variation analysis (GSVA) was employed to quantify the involvement of BP in each sample using “GSVA” package of the R software [18]. The “limma” package was further employed to calculate the differentially enriched BP between C1 and C2 [14]. The cutoff values were as follows: ∣log_2_FC | >0.5 and adj.*p*.val < 0.05.

### 2.5. Immune Microenvironment Estimation

The immune microenvironment estimation was conducted by ESTIMATE, ssGSEA, MCPcounter, QUANTISEQ, and TIMER algorithms [19–23]. The association between hub genes and immune infiltration was also analyzed by applying TIMER 2.0 database [23]. Tumor immune dysfunction and exclusion (TIDE) algorithm was used to predict the response to ICIs [24].

### 2.6. Construction and Validation of a Risk Score

According to patients' survival, LASSO algorithm was employed to establish a risk score in TCGA dataset, which was achieved by the “lars” package of R [25]. Subsequently, samples were divided into low- and high-risk groups. Survival analysis was achieved by the “survival” package. In addition, its prognostic value was validated in GSE62254 and GSE84437 datasets. As for the hub genes, their association with GC survival was estimated by the Kaplan-Meier Plotter online tool [26].

### 2.7. Somatic Mutation Analysis

To investigate the somatic mutation in GC, the tumor mutational data were downloaded from TCGA and analyzed by the “maftools” package of R [27]. TMB was also calculated in each GC sample from TCGA.

### 2.8. Relationship between M6A Genes and Risk Score

M6A regulatory genes are comprised of readers (*YTHDC1*, *YTHDC1*, *YTHDF1*, *YTHDF2*, *YTHDF3*, *HNRNPC*, *HNRNPA2B1*, *IGF2BP1*, *IGF2BP2*, *IGF2BP3*, *FMR1*), writers (*METTL3*, *METTL14*, *METTL16*, *WTAP*, *METL16*, *RBM15*, *RBM15B*, *VIRMA*, and *ZC3H13*) and erasers (*ALKBH3*, *ALKBH5*, and *FTO*) [28]. The association between M6A regulatory genes and risk score was accessed by Pearson correlation analysis and visualized by “ggplot2” package of R.

### 2.9. Statistical Analysis

The R software (version 4.1.0) or IBM SPSS Statistics 23 was used to conduct statistical analyses. The *t*-test was applicable to normally distributed data and Mann–Whitney test to nonnormally distributed data. Survival time represented the time from diagnosis to the last follow-up or death. Unless otherwise specified, *p* < 0.05 was considered as a statistically significant result.

## 3. Results

### 3.1. NF-*κ*B Signaling Pathway Was Significant Association with Immune Infiltration

**Figure 2:**
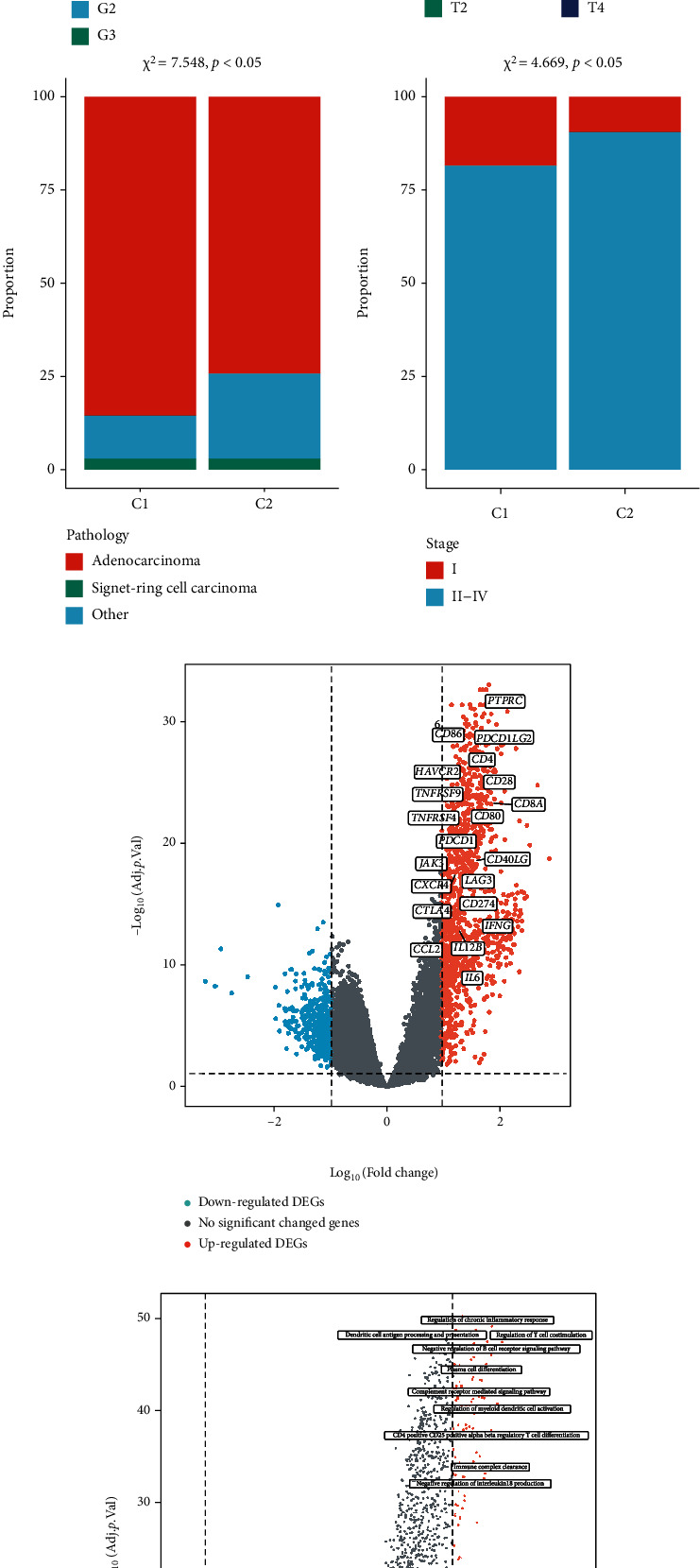
GC samples were clustered by NF-*κ*B signaling pathway score. (a) The expression of NF-*κ*B-related genes in GC samples. (b) GC samples were divided into two cluster (C1 and C2 type) based on NF-*κ*B signaling pathway score (setting median value as cutoff). The bar charts showed the proportions of different grade, T, pathology, and stage in C1 and C2 type. (c) Volcano plot showed the DEGs between C1 and C2 type. The labeled items were immune checkpoint-related genes. (d) Volcano plot showed differential biological processes analyzed by GSVA between C1 and C2 type. (e) GSEA showed that multiple immune-related biological processes were enriched in C2 type. GC: gastric cancer; DEGs: differential expressed genes; GSVA: gene set variation analysis; GSEA: gene set enrichment analysis.

At first, NF-*κ*B-related genes (*n* = 104) were obtained from the KEGG database and ssGSEA algorithm was applied to calculating each sample's NF-*κ*B signaling pathway score in TCGA dataset. The heatmap showed the expression of these NF-*κ*B-related genes in GC (Figure 2(a)). According to the median value, GC samples were classified into low (C1) and high (C2) NF-*κ*B signaling pathway groups. Compared to C1 type characterized by low enrichment of NF-*κ*B signaling pathway, C2 type had more high-grade samples, more advanced cases, and less adenocarcinoma proportion (Figure 2(b)), indicating that NF-*κ*B signaling pathway was involved in a worse phenotype. The DEGs between C1 and C2 were shown in the volcano plot (Figure 2(c)). Among that, we found that more immune checkpoint-related genes, such as *CD274* (PD-L1), *CTLA4*, and *LAG3*, enriched in C2 type GC. GSVA was next applied to analyze the differential biology processes between C1 and C2 type GC. The data indicated that the major enriched biology processes were related to cancer immunity (Figure 2(d)). GSEA also demonstrated that these DEGs mainly enriched in immune-related biology processes (Figure 2(e)). These data suggested that the NF-*κ*B signaling pathway was closely concerned in GC's immune microenviroment, being consistent with previous studies [4, 6, 7].

**Figure 3:**
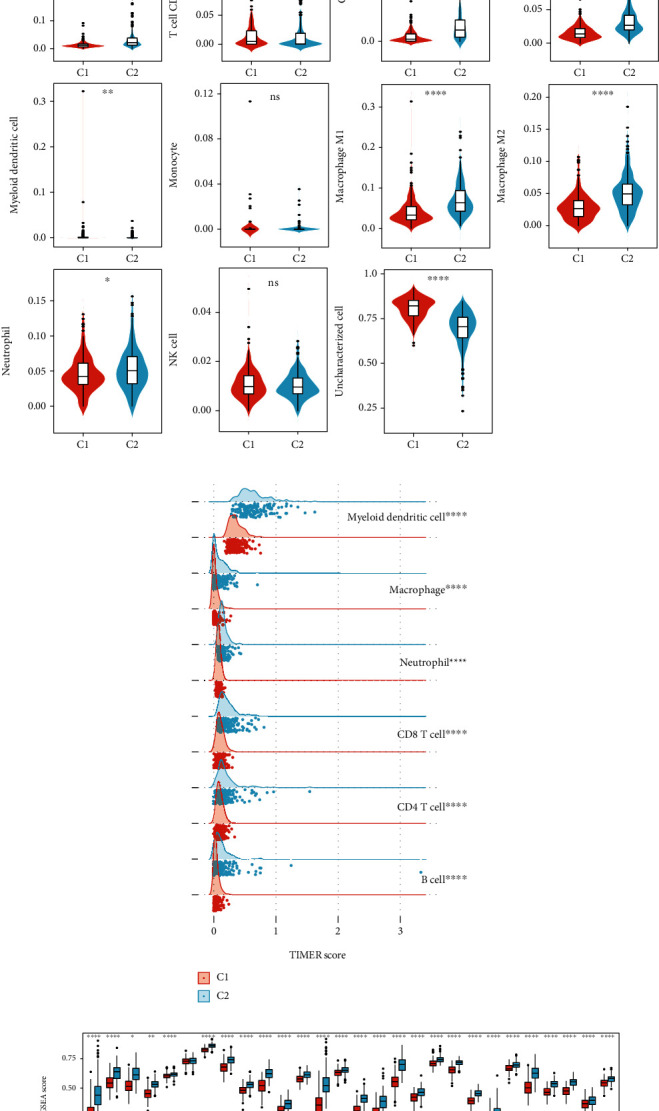
C2 type had a high immune infiltration. The immune infiltration was evaluated by (a) ESTIMATE, (b) QUANTISEQ, (c) TIMER, and (d) ssGSEA algorithms. ^∗^*p* < 0.05, ^∗∗^*p* < 0.01, ^∗∗∗^*p* < 0.001, and ^∗∗∗∗^*p* < 0.0001.

For this reason, we next evaluated the association between NF-*κ*B signaling pathway score and immune infiltration in TCGA dataset. C2 type had a higher immune score, stromal score, and ESTIMATE score, but a lower tumor purity than C1 (Figure 3(a)). The results of QUANTISEQ algorithm showed that C2 had higher B cells (*p* < 0.0001), CD8 T cells (*p* < 0.0001), Tregs (*p* < 0.0001), M1 macrophage (*p* < 0.0001), M2 macrophage (*p* < 0.0001), and neutrophils (*p* < 0.05), while C1 had higher uncharacterized cells (*p* < 0.0001) (Figure 3(b)). Further, TIMER and ssGSEA algorithms found that all estimated immune cells in C2 were significantly higher than that in C1 (Figures 3(c) and 3(d)). These results implied that patients with high level of NF-*κ*B signaling pathway had a high infiltration of immune cells.

### 3.2. Establishing a Risk Score Based on NF-*κ*B-Related Genes

**Figure 4:**
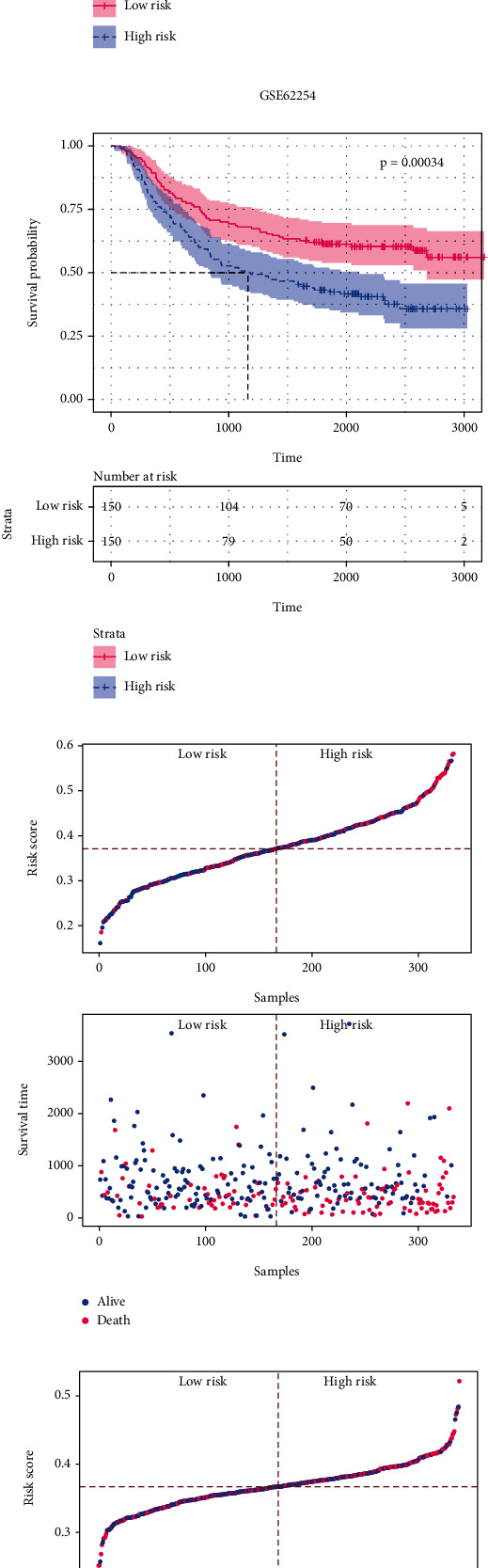
Construction of a seven-gene risk score. (a) Volcano plot showed the DEGs between normal and tumor samples in TCGA. (b) Venn plot showed the overlapping genes between DEGs and NF-*κ*B-related genes. (c) The parameters of LASSO algorithm. (d–f). Survival analysis of the risk score in (d) discovery cohort and (e, f) validation cohort. (g–i). Risk score, survival status, survival time in (g) TCGA, (h) GSE84437, and (i) GSE62254 dataset. (j–l). The expression of the seven hub genes in (j) TCGA, (k) GSE84437, and (l) GSE62254 dataset. DEGs: differentially expressed genes; TCGA: The Cancer Genome Atlas; LASSO: least absolute shrinkage and selection operator.

Next, a total of 10451 DEGs (9043 upregulated and 1408 downregulated genes) were identified between tumor and normal samples in TGCA dataset (Figure 4(a)). The Venn diagram showed the overlapped genes between these DEGs and NF-*κ*B-related genes, consisting of 31 upregulated and 3 downregulated genes (Figure 4(b)). Based on these genes, LASSO algorithm was used to construct a seven-gene risk score as follows: risk score = (0.0211 × Exp *CARD*11) + (0.0005 × Exp *CCL*21) + (0.0337 × Exp *GADD*45*B*) + (0.0212 × Exp *LBP*) + (–0.0211 × Exp *RELB*) + (–0.0075 × Exp *TRAF*2) + (0.0009 × Exp *VCAM*1) (Figure 4(c)). Survival analysis revealed that samples with high risk score displayed a poorer overall survival (OS) than those with low risk score (median OS, mOS 779*vs*. 1407 days, *p* = 0.005; Figure 4(d)). The high-risk group also showed a shorter OS than the low-risk group in both validation cohort GSE84437 (*p* = 0.023) and GSE62254 (*p* = 0.00034; Figures 4(e) and 4(f)). The mOS of the high-risk group was 2610 and 1162 days in these two cohorts, respectively, while the mOS of the low-risk group has not reached within the follow-up time. With increasing risk score, the probability of poor prognosis was increased in both discovery and validation queues (Figures 4(g)–4(i)). Followed heatmap showed the expression of these seven hub genes (Figures 4(j)–4(l)). In addition, the high-risk group had a higher level of NF-*κ*B signaling pathway score than the low-risk group (Figure S2). Previous studies have put forward several risk score models in GC [29–32]. These risk score models were further analyzed in TCGA cohort (Table S2), and their efficacy was estimated by receiver operating characteristic curve (ROC) and area under the curve (AUC). The results showed that our seven-gene risk score had the highest AUC value (Figure S3A). Collectively, a NF-*κ*B-related risk score was constructed for predicting GC patients' prognosis.

### 3.3. High-Risk Group Had High Immune Infiltration but Low Response to Immunotherapy

**Figure 5:**
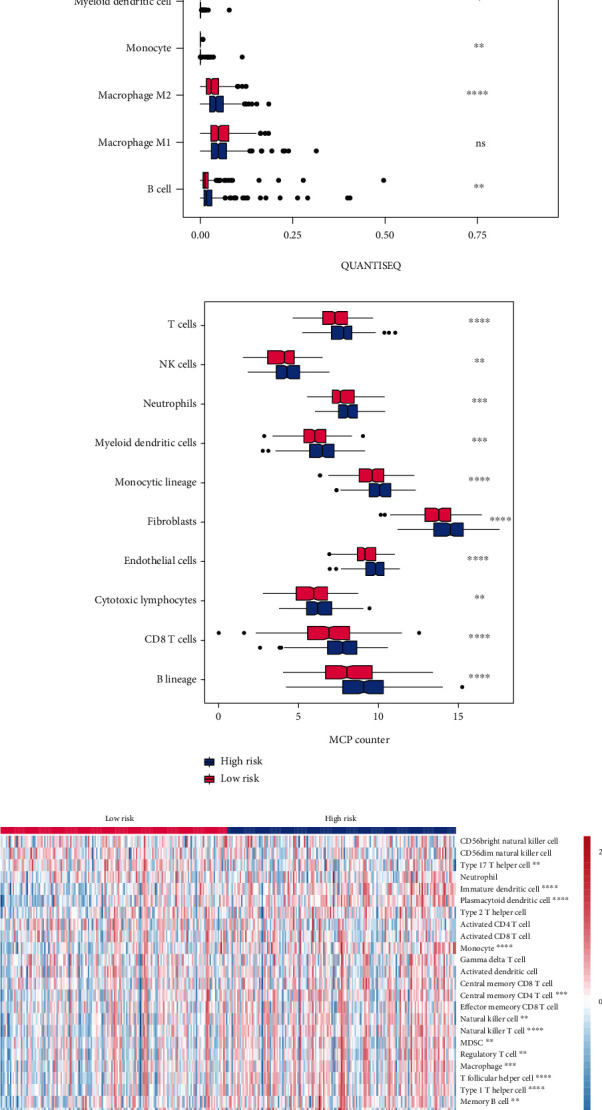
High-risk group possessed a high immune infiltration and TIDE score. (a–d) The immune infiltration was evaluated by (a) ESTIMATE, (b) QUANTISEQ, (c) MCPcounter, and (d) ssGSEA algorithms between low- and high-risk groups. (e) TIDE score in low- and high-risk groups. (f) The proportion of patients who may response to ICIs in low- and high-risk groups. TIDE: tumor immune dysfunction and exclusion; ssGSEA: single-sample gene set enrichment analysis; immune checkpoint inhibitors: ICIs; ^∗^*p* < 0.05, ^∗∗^*p* < 0.01, ^∗∗∗^*p* < 0.001, and ^∗∗∗∗^*p* < 0.0001.

Then, the immune infiltration was estimated between the two risk groups. Obviously, the high-risk group had a higher stromal score (*p* < 0.0001), immune score (*p* < 0.05), and ESTIMATE score (*p* < 0.001) compared with the low-risk group (Figure 5(a)). Instead, its tumor purity was lower than the low-risk group (*p* < 0.001). The correlation was estimated between the seven-gene risk score and immune score, stromal score, and ESTIMATE score as well as tumor purity. The results showed that only the seven-gene risk score was significant association with all of the four scores (Figure S3B). QUANTISEQ algorithm indicated that the high-risk group possessed more immune cell infiltration, including B cells (*p* < 0.01), M2 macrophage (*p* < 0.0001), monocyte (*p* < 0.01), Tregs (*p* < 0.01), CD8 T cells (*p* < 0.001), and nonregulatory CD4 T cells (*p* < 0.01), but less uncharacterized cells (*p* < 0.001) compared with the low-risk group (Figure 5(b)). MCPcounter algorithm could calculate the absolute abundance of eight immune cells (T cells, NK cells, neutrophils, myeloid dendritic cells, monocytic lineage, cytotoxic lymphocytes, CD T cells, and B lineage) and two stromal cells (fibroblasts and endothelial cells) in heterogeneous tissues from transcriptomic data [21]. Our data showed that all of those cell populations had a more significant infiltration in the high-risk group than in the low-risk group (Figure 5(c)). Further, ssGSEA algorithm reinforced the high level of immune infiltration in the high-risk group (Figure 5(d)). In a word, the high-risk group had a higher infiltration of immune cells than the low-risk group.

Unfortunately, high immune infiltration has not brought a favourable outcome for the high-risk group, implying higher probability of immune evasion. To this end, we evaluated TIDE score—an estimation of two distinct immune evasion mechanisms in tumor, that is dysfunction of tumor infiltration cytotoxic T lymphocytes (CTL) and immunosuppressive factors drove exclusion of CTL, between the two groups [24]. Compared to the low-risk group, the high-risk group had a higher TIDE score (*p* < 0.01), indicating more probabilities of immune evasion occurred in this group (Figure 5(e)). Of note, the low-risk group had a higher predicted response to ICIs than the high-risk group (47.59% *vs*. 28.14%, *p* < 0.001; Figure 5(f)).

### 3.4. High-Risk Group Possessed Low Tumor Mutational Burden

**Figure 6:**
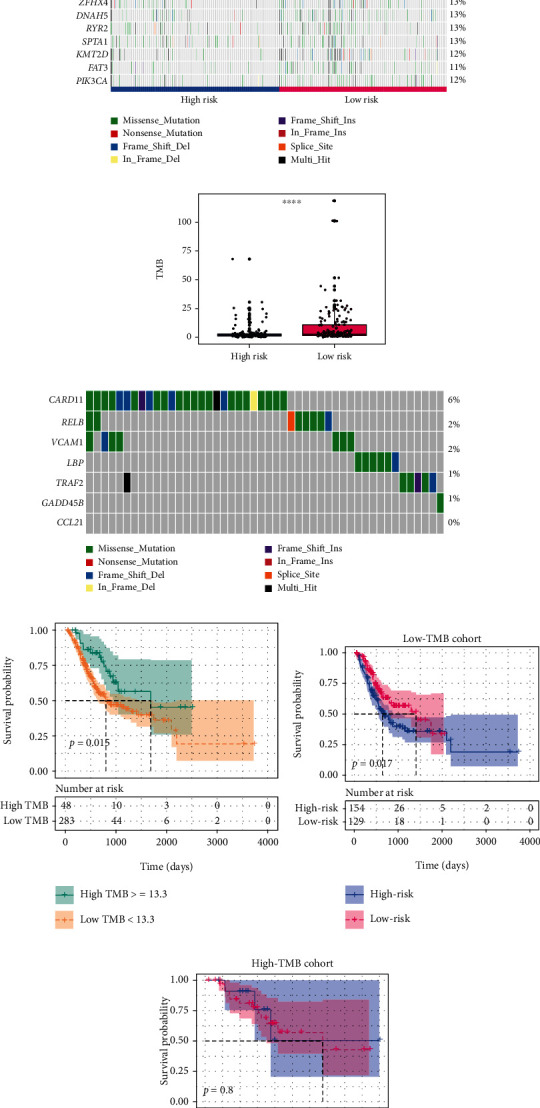
High-risk group had a low TMB. (a) The waterfall plot showed the top 20 frequent mutations that were occurred in GC. (b) The low-risk group had a higher TMB than the high-risk group. (c) The mutations of the seven hub genes in GC. (d) Survival analysis of TMB in GC. Samples were divided into low- and high-TMB groups according to the optimal cutoff value. (e, f) Stratified analysis showed that the high-risk group tended to a poorer outcome than the low-risk group in low-TMB cohort, but not in high-TMB cohort. TMB: tumor mutational burden; GC: gastric cancer. ^∗∗∗∗^*p* < 0.0001.

Theoretically, the more mutations, the more neoantigens, the higher probabilities for T cell recognition, and the better ICI response [33]. Here, GC mutational data was obtained from TCGA database to evaluate somatic mutations. We found a higher TMB in the low-risk group, which may be the reason for its higher response rate to ICIs (Figures 6(a) and 6(b)). We also investigated the mutation of the seven hub genes in GC and observed that these genes displayed low mutational rate in GC, ranging from 0% to 6% (Figure 6(c)). Then, patients were divided into low- and high-TMB cohorts based on the optimal cutoff value calculated by “survival” package (Figure S3). Results showed that patients with high TMB have a better prognosis than those with low TMB (1686 *vs*. 801 days, *p* = 0.015; Figure 6(d)). Further stratified analysis indicated that the low-risk group has a significant longer OS than the high-risk group in low-TMB (mOS 1407 *vs*. 640 days; *p* = 0.017) cohort, but no significant difference was observed in the high-TMB cohort (*p* = 0.8; Figures 6(e) and 6(f)), which may be due to the small samples in the high-TMB cohort. These data suggested that low TMB in high-risk score may be an another factor that caused its poor outcome.

### 3.5. Association between the Risk Score and M6A

**Figure 7:**
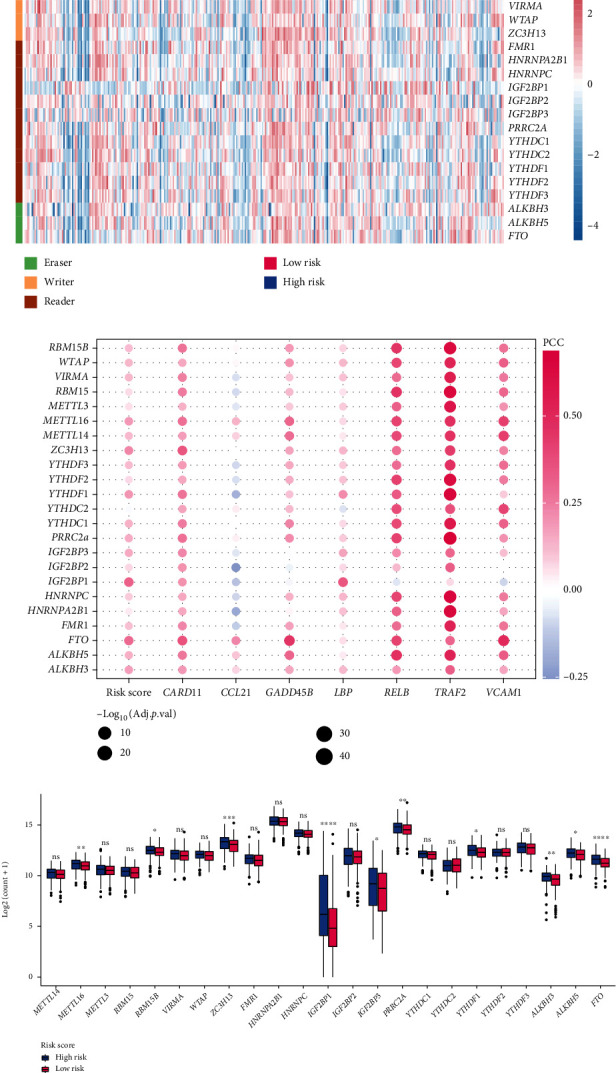
The expression of M6A regulatory genes in low- and high-risk groups. (a) Heatmap showed the expression of M6A regulatory genes in GC sample from TCGA. (b) The correlation between M6A regulatory genes and the risk score, as well as seven hub genes. (c) The expression of the M6A regulatory genes in low- and high-risk groups. M6A: N6-methyladenosine; GC: gastric cancer; TCGA: The Cancer Genome Atlas; ^∗^*p* < 0.05, ^∗∗^*p* < 0.01, ^∗∗∗^*p* < 0.001, and ^∗∗∗∗^*p* < 0.0001.

As a reversible epigenetic modification, M6A could affect both messenger RNA and noncoding RNAs in eukaryotes and play crucial roles in diverse cancer pathological processes, including immune evasion [28]. M6A regulatory genes are divided into writers, erasers, and readers, which are often dysregulated in various cancer types [28]. The heatmap showed the expression of common M6A regulatory genes in high- and low-risk groups (Figure 7(a)). The results of Pearson correlation analysis showed that the risk score was positively correlated with the expression of most M6A regulatory genes, such as *IGF2BP1*, *FTO*, and *ZC3H13* (Figure 7(b)). The expression of the seven hub genes also significantly related to the expression of M6A regulatory genes, and the majority of which was positive correlation. Among that, *TRAF2* was most correlated with M6A regulatory genes. Further analysis indicated that the high-risk group had higher expression of *METTL16* (*p* < 0.01), *RBM15B* (*p* < 0.05), *ZC3H13* (*p* < 0.001), *IGF2BP1* (*p* < 0.0001), *PRRC2A* (*p* < 0.05), *YTHDF1* (*p* < 0.01), *ALKBH3* (*p* < 0.01), *ALKBH5* (*p* < 0.05), and *FTO* (*p* < 0.0001) compared to the low-risk group (Figure 7(c)). These results suggested the close implication of NF-*κ*B signaling pathway in M6A regulatory genes.

### 3.6. Most of Hub Genes Had an Independent Prognostic Significance

**Figure 8:**
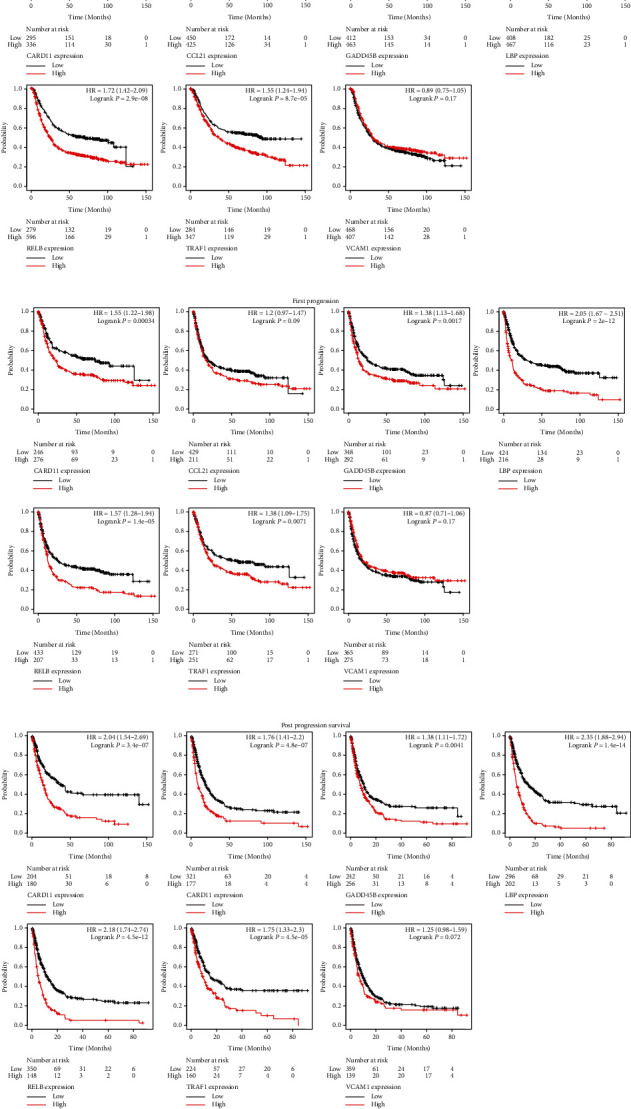
The correlation between the hub genes and prognosis of patients. (a) The correlation between the expression of the seven hub genes and OS in GC. (b) The correlation between the expression of the seven hub genes and FP in GC. (c) The correlation between the expression of the seven hub genes and PPS in GC. OS: overall survival; GC: gastric cancer; FP: first progression; PPS: postprogression survival; HR: hazard ratio.

Subsequently, we evaluated the potential prognostic value of these seven hub genes in GC through retrieving Kaplan-Meier Plotter online tool. As shown, nearly all the hub genes displayed a prognostic significance for GC patients except *VCAM1* (Figure 8(a)). In particular, high expression of *CARD11* had a shorter OS than low expression (mOS, 32.6 *vs*. 93.2 months; *p* < 0.0001), which was consistent with the predictive significance of other five hub genes, including *CCL21* (mOS, 26.8 *vs*. 36.2 months; *p* < 0.01), *GADD45B* (mOS, 26.3 *vs*. 35.4 months; *p* < 0.001), *LBP* (mOS, 22.0 *vs*. 57.6 months; *p* < 0.0001), *RELB* (mOS, 23.9 *vs*. 65.0 months; *p* < 0.0001), and *TRAF1* (mOS, 36.4 *vs*. 93.2 months; *p* < 0.0001). In addition, patients with high *CARD11* had shorter time of first progression (FP) and postprogression survival (PPS) than those with low *CARD11* (Figures 8(b) and 8(c)). Similar results were observed when analyzing *CCL21*, *GADD45B*, *LBP*, *RELB*, and *TRAF1*, but not *VCAM1*. These data indicated that the hub genes other than *VCAM1* may be an independent prognostic signature for GC.

### 3.7. Most of Hub Genes Were Closely Association with Immune Infiltration

**Figure 9:**
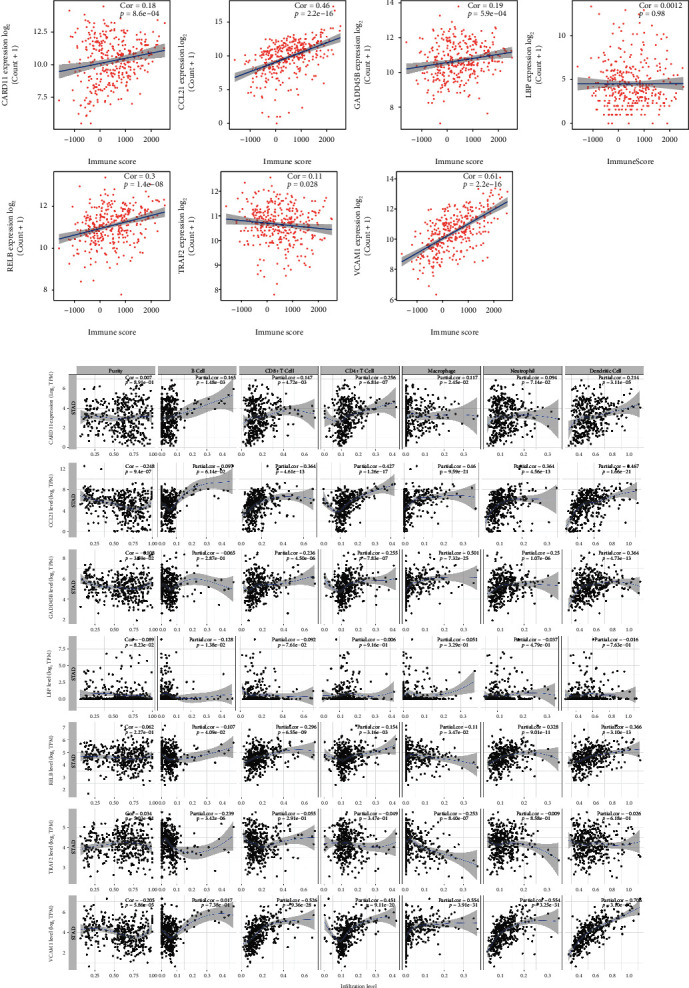
The involvement of the hub genes in immune infiltration. (a) The correlation between the seven hub genes and immune score calculated by ESTIMATE algorithm in GC. (b) The correlation between the seven hub genes and immune infiltration investigated by TIMER2.0 database.

Finally, the correlation between these hub genes and immune infiltration was investigated. The results revealed that the hub genes except *LBP* were positively correlated with the immune score (Figure 9(a)). Further data indicated the significant association of these hub genes with immune cell infiltration (Figure 9(b)). In detail, infiltration of B cells was negatively correlated with *LBP*, *RELB*, and *TRAF2* but positively correlated with *CARD11*; infiltration of CD8 T cells, CD4 T cells, and dentritic cells was positively associated with *CARD11*, *CCL21*, *GADD45B*, *RELB*, and *VCAM1*; macrophages' infiltration was positively correlated with *CARD11*, *CCL21*, *GADD45B*, *RELB*, and *VCAM1*, while negatively correlated with *TRAF2*; neutrophils' infiltration was positively associated with *CCL21*, *GADD45B*, *RELB*, and *VCAM1*. In summary, these hub genes may play an important role in regulating GC's immune microenvironment.

## 4. Discussion

In this work, ssGSEA algorithm was applied to calculating a NF-*κ*B signaling pathway score. We found this score was significantly associated with immune infiltration in GC. In addition, a seven-gene risk score was established according to NF-*κ*B-related genes. High risk score group had a higher immune infiltration and M6A level, but a lower TMB, compared to the low risk score group. Further results indicated that the high risk score group tended to an immunosuppressive microenviroment and showed a poor response to ICIs. Finally, most of the hub genes (*CARD11*, *CCL21*, *GADD45B*, *LBP*, *RELB*, *TRAF1*, and *VCAM1*) had an independent prognostic signature and performed a close connection to immune infiltration in GC.

Deciphering the molecular mechanism of immune evasion in cancer is the lynchpin to achieving the goal of tailored immunotherapy. As abovementioned, NF-*κ*B signaling pathway worked in almost all of the infiltrated cells in tumor microenvironment [34–36]. For instance, NF-*κ*B participates in macrophage polarization and transforms them from a tumor-promoting M2 phenotype to a M1-like cytotoxic phenotype [34]; NF-*κ*B plays an essential role in T cell and B cell activation, as well as development [35, 36]. In GC, IL-1beta activated MDSCs through an IL-1RI/NF-*κ*B pathway, contributing to an immunosuppressive microenvironment, and whereby promoted tumor progress [37]. Our results showed that the high enrichment of NF-*κ*B signaling pathway tends to increase the infiltration of various immune cells. These data indicated that NF-*κ*B signaling pathway played an essential role in GC immune microenvironment. We next constructed a seven-gene risk score based on NF-*κ*B-related genes and divided the samples into low- and high-risk groups with the median as cutoff value. As expected, low- and high-risk groups also displayed distinct immune infiltration. However, high immune infiltration did not bring about a better prognosis in GC. Analyzing the subtype of the infiltrated immune cells, we found that both immunosuppressive (e.g., Tregs, MDSCs, and macrophage M2) and immunostimulative (CD8 T cells, CD4 T cells, and NK cells) cells were significantly enriched in the high-risk group. Of note, the high-risk group possessed a higher TIDE score, indicating more dysfunctional anticancer immune cells and higher possibility of immune evasion in this group [24]. Accordingly, although NF-*κ*B signaling pathway brings more infiltration of immune cells, it may contribute to an immunosuppressive microenvironment in GC, leading to a worse outcome.

Another observation further explained the reason of the poor prognosis in the high-risk group, that is, lower TMB in this group. TMB is considered as a predictive biomarker of response to ICIs, which has been verified retrospectively or prospectively in melanoma, lung cancer, bladder cancer, *etc*. [38]. Some study revealed that TMB could also be a predictive biomarker for predicting the efficacy of chemotherapy and target therapy [39, 40]. This study agreed that high TMB predicts a better therapeutic response. A systematic pan-cancer analysis estimated the general prognostic impact of TMB in patients with solid tumors based on TCGA database and revealed that the predictive value of TMB varies from different cancer types [41]. Patients with high TMB had a significantly longer OS than those with low TMB (*p* = 0.003) in GC [41], being consistent with our data. Taken together, high TMB in the low-risk group may be another factor for their superior prognosis. In addition, the TIDE algorithm indicated the higher response rate to ICIs in the low-risk group. Although the different TMB may be a major determination factor to this phenomenon, the risk score was also considered as a potential biomarker for predicting response to ICIs in GC.

As a dynamic and reversible methyl-modification, increasing evidences indicated the closely connection between M6A and immune infiltration characterization in various cancers, including GC [42, 43]. M6A modification is mainly regulated by its related genes, which can be classified into readers, writers, and erasers. NF-*κ*B signaling pathway transcriptionally regulated the expression of a broad range of target genes. We found our risk score was significantly associated with several M6A regulatory genes. What is more, the expression of *METTL16*, *RBM15B*, *ZC3H13, IGF2BP1*, *PRRC2A*, *YTHDF1*, *ALKBH3*, *ALKBH5*, and *FTO* was significantly upregulated in the high-risk group than in the low-risk group. These genes played a crucial role in tumor microenviroment. For instance, *ALKBH5* regulated Tregs and MDSC accumulation via modulating the expression of Mct4/Slc16a3 [44]; *FTO* facilitated immune invasion and desensitized tumor cells to T cell cytotoxicity [45]; *YTHDF1* was correlated with immune cell infiltration but attenuated DCs' cross-presentation capacity [46, 47]. Therefore, NF-*κ*B signaling pathway may regulate GC's immune infiltration via affecting M6A modification.

We next focused on the seven NF-*κ*B related genes (*CARD11*, *CCL21*, *GADD45B*, *LBP*, *RELB*, *TRAF1*, and *VCAM1*). As well known, NF-*κ*B signaling pathway played a crucial role in Helicobacter Pylori-related gastric carcinogenesis and progression. Its related genes, *TRAF1*, *VCAM1*, and *RELB*, also participated in this progress [48–50]. In addition, CCL21 worked as a driver factor via MALAT1/SRSF1/mTOR axis during the progression of GC [51]. Nevertheless, little study revealed the function of *CARD11*, *GADD45B*, and *LBP* in GC. Here, we found that except *LBP*, these hub genes performed significant correlation with immune infiltration in GC. Previous study also indicated the role of these genes played in immune function [52–54], which has not been reported in GC yet. To this end, study of these genes may deepen our understanding of the mechanism underlying GC's immune microenviroment.

## 5. Conclusions

In summary, a clear correlation was revealed between GC immunity and NF-*κ*B signaling pathway, as well as the risk score based on NF-*κ*B-related genes. We also found that NF-*κ*B signaling pathway was significant association with TMB as well M6A level in GC. In addition, most of hub genes performed an independent prognosis value and significant correlation with immune infiltration in GC. These results indicated that NF-*κ*B signaling pathway played a crucial role in GC immunity, and M6A modification may be an important bridge between them. However, only bioinformatics analyses are not enough to clear the mechanisms underlying NF-*κ*B signaling pathway mediated cancer immunity, and further experimental works are necessary. Accordingly, identified hub genes will be the focus in the follow-up study, which may deepen the understanding of cancer immunity and provide a novel strategy for immunotherapy in GC.

## Data Availability

All data were obtained from TCGA, GEO, KEGG, Kaplan-Meier online tool, and TIMER 2.0 datasets.

## Supplementary Materials

Supplementary Materials Figure S1: the genes enriched in NF-*κ*B signaling pathway. Figure S2: NF-*κ*B signaling pathway score in different risk score groups. Figure S3: comparison of the seven-gene risk score and previous risk score in GC. Figure S4: the cutoff value of TMB in TCGA cohort. Table S1: the clinical characteristics of GC samples in TCGA and GEO. Table S2: the risk score of each model in GC.
