# Supplementary material for: Bioinformatics Analysis Revealing the Correlation between NF-κB Signaling Pathway and Immune Infiltration in Gastric Cancer
Source: Comput Math Methods Med. 2022 Jul 28;2022:5385456. doi: 10.1155/2022/5385456 (PMC9352505; doi:10.1155/2022/5385456)
Supplement: Supplementary Materials — Figure S1: the genes enriched in NF-κB signaling pathway. Figure S2: NF-κB signaling pathway score in different risk score groups. Figure S3: comparison of the seven-gene risk score and previous risk score in GC. Figure S4: the cutoff value of TMB in TCGA cohort. Table S1: the clinical characteristics of GC samples in TCGA and GEO. Table S2: the risk score of each model in GC. [file 5385456.f1.docx]

**Figure S1 The genes enriched in NF-κB signaling pathway.**


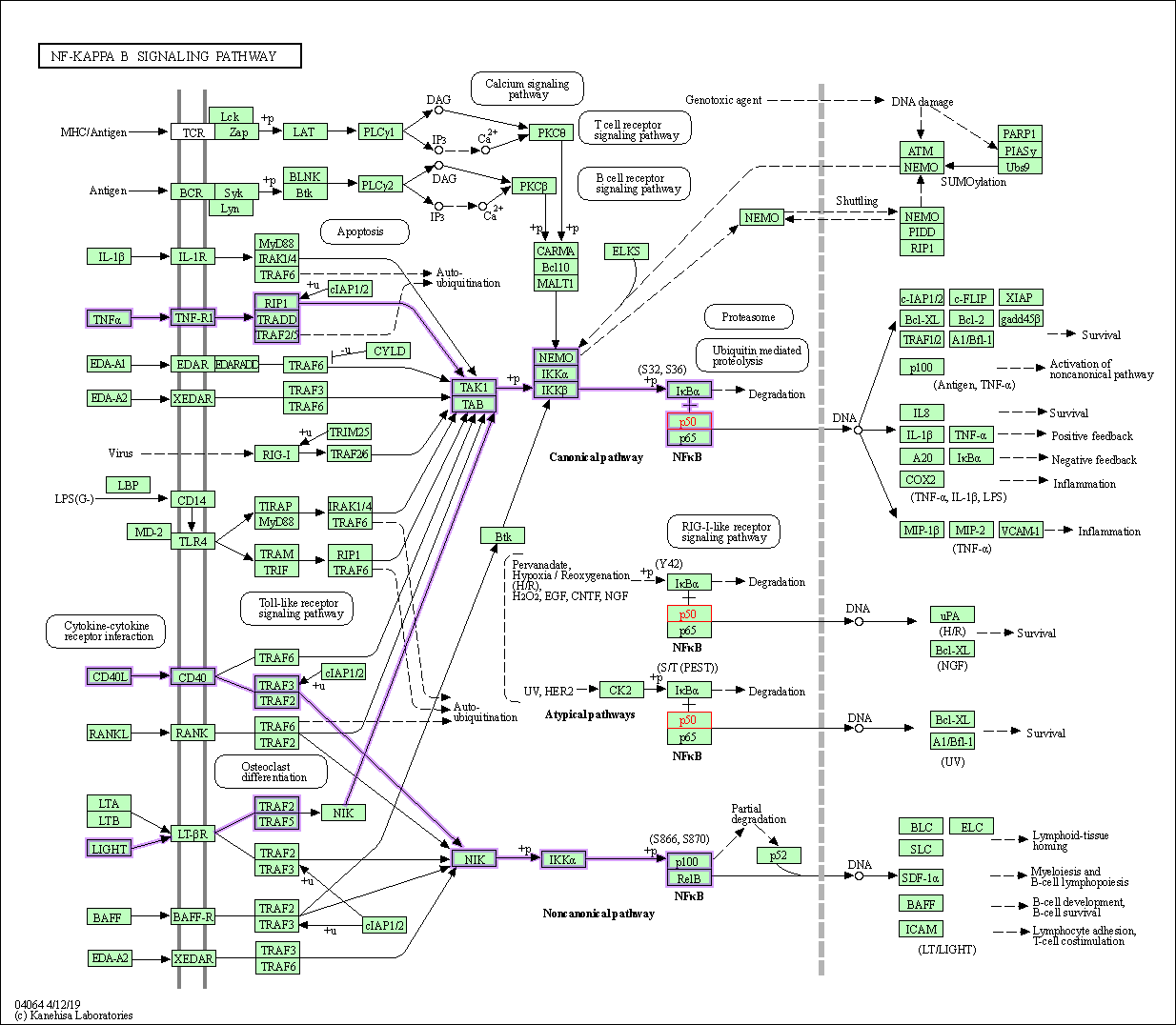


**Figure S2 NF-κB signaling pathway score in different risk score groups.**


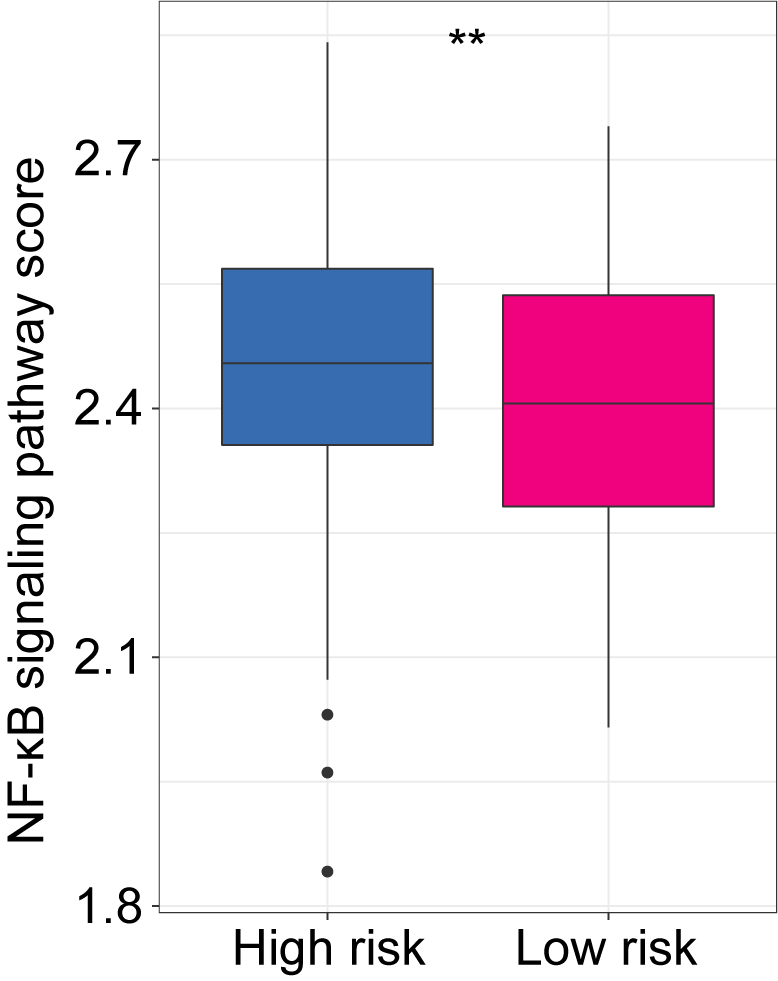


**Figure S3 Comparison of the seven-gene risk score and previous risk score in GC.** A. The ROC of each risk score in TCGA cohort. B. The correlation between each risk score and immune score in TCGA cohort.


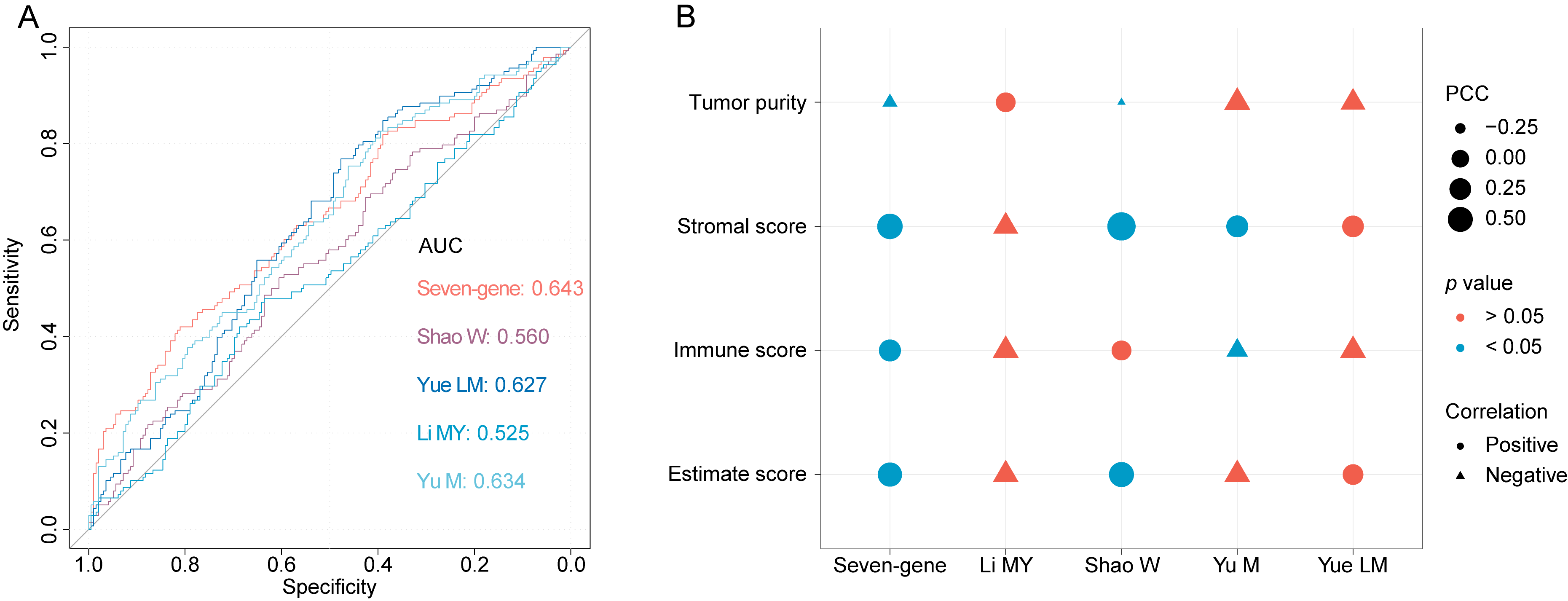


**Figure S4 The cutoff value of TMB in TCGA cohort.**


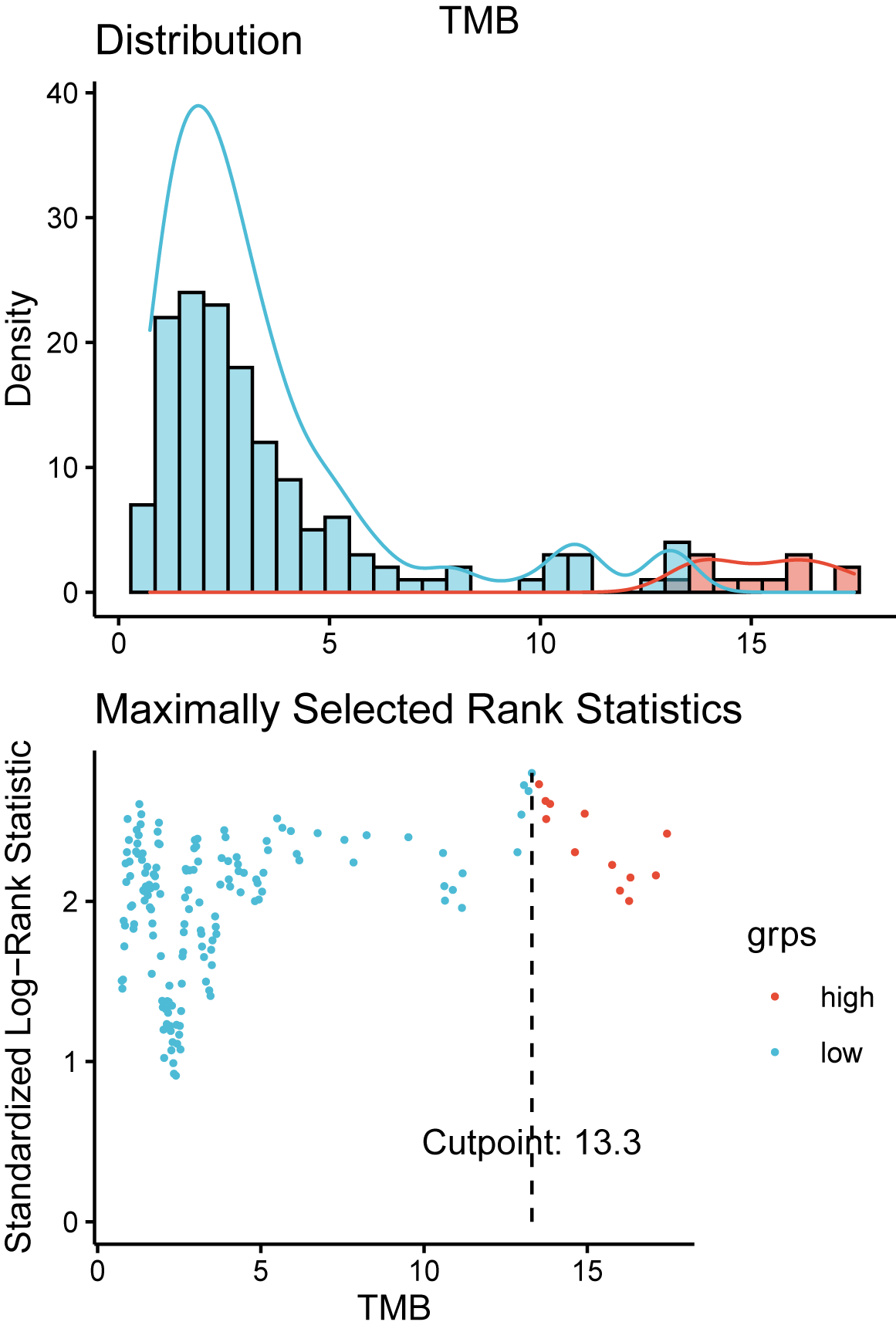


**Table S1 Characteristics of GC patients in TCGA and GEO datasets.**

|  | **TCGA**  **(n = 333)** | **GSE62254**  **(n = 300)** | **GSE84437**  **(n = 433)** |
| --- | --- | --- | --- |
| **Age** |  |  |  |
| <= 65 | 153 | 172 | 283 |
| > 65 | 180 | 128 | 150 |
| **Sex** |  |  |  |
| male | 215 | 199 | 296 |
| female | 118 | 101 | 137 |
| **Grade** |  |  |  |
| G1 | 9 |  |  |
| G2 | 119 |  |  |
| G3 | 197 |  |  |
| **Stage** |  |  |  |
| I | 45 | 30 |  |
| II | 105 | 97 |  |
| III | 136 | 96 |  |
| IV | 34 | 77 |  |
| **Pathology** |  |  |  |
| adenocarcinoma | 266 | 223 |  |
| signet ring cell carcinoma | 10 | 42 |  |
| other | 57 | 35 |  |

GC, gastric cancer; The Cancer Genome Atlas, TCGA; Gene Expression Omnibus, GEO.

**Table S2. The risk score of each model in GC.**

| **ID** | **Seven-gene** | **Shao W** | **Li MY** | **Yue LM** | **Yu M** |
| --- | --- | --- | --- | --- | --- |
| TCGA-3M-AB46 | 0.276959354 | 3.840235793 | 0.039674313 | 4.128332094 | 3.928327128 |
| TCGA-B7-5818 | 0.291072592 | 2.966946841 | 0.639802103 | 4.159371216 | 4.374403746 |
| TCGA-B7-A5TI | 0.357080952 | 5.340689315 | 0.619417566 | 3.606532188 | 5.31260246 |
| TCGA-B7-A5TJ | 0.357254924 | 5.436839015 | 0.998383344 | 4.664842859 | 6.70265651 |
| TCGA-B7-A5TK | 0.466568892 | 8.531590461 | -0.308914968 | 3.707033484 | 6.098725431 |
| TCGA-B7-A5TN | 0.438099877 | 5.023749041 | 0.454010552 | 4.243923454 | 4.503936549 |
| TCGA-BR-4187 | 0.528223857 | 8.635019942 | -1.094231874 | 4.203040884 | 7.317242844 |
| TCGA-BR-4191 | 0.441033751 | 5.094254625 | 0.589103459 | 4.70865488 | 5.83838322 |
| TCGA-BR-4201 | 0.441751208 | 5.868384923 | -0.805993956 | 4.060249208 | 6.313398062 |
| TCGA-BR-4253 | 0.41019677 | 2.265254275 | 0.870669291 | 4.34223189 | 5.340830924 |
| TCGA-BR-4256 | 0.483080885 | 8.555253376 | 0.355863629 | 5.053178945 | 7.233936273 |
| TCGA-BR-4257 | 0.329333812 | 3.237669185 | 0.433312721 | 4.443722772 | 4.896712691 |
| TCGA-BR-4267 | 0.486787821 | 4.482147954 | 1.656865268 | 3.658633453 | 5.455262883 |
| TCGA-BR-4279 | 0.49741926 | 7.379461607 | -0.166234427 | 3.514569125 | 6.512629583 |
| TCGA-BR-4280 | 0.294961722 | 2.541556998 | 0.156667661 | 4.380407972 | 4.505413881 |
| TCGA-BR-6452 | 0.277713197 | 4.209402284 | 0.386772928 | 4.2381761 | 4.625919649 |
| TCGA-BR-6453 | 0.418002825 | 5.184043239 | -0.59385659 | 3.737155046 | 5.359371477 |
| TCGA-BR-6455 | 0.359564676 | 6.004329221 | -0.413967037 | 3.928516311 | 6.055303059 |
| TCGA-BR-6456 | 0.494531531 | 8.393855306 | 0.313996956 | 4.16777369 | 4.105283649 |
| TCGA-BR-6457 | 0.462532918 | 9.626307555 | 0.225879455 | 4.710727246 | 5.664979706 |
| TCGA-BR-6458 | 0.390635633 | 6.08401838 | 0.899992203 | 3.335681855 | 5.420909005 |
| TCGA-BR-6563 | 0.38884859 | 7.464924124 | 1.086558306 | 4.201675328 | 5.093962356 |
| TCGA-BR-6564 | 0.422377581 | 10.82229401 | 2.049432608 | 3.143954816 | 5.041854028 |
| TCGA-BR-6565 | 0.34382201 | 6.602701246 | -0.298737691 | 4.225002725 | 3.514044883 |
| TCGA-BR-6566 | 0.254227906 | 3.683309183 | 0.30076886 | 3.671292173 | 5.346982651 |
| TCGA-BR-6705 | 0.537508041 | 7.929642769 | -0.911517585 | 4.095696776 | 6.815179032 |
| TCGA-BR-6707 | 0.263277529 | 3.903474169 | 1.833484479 | 4.433971416 | 3.874689275 |
| TCGA-BR-6709 | 0.293608611 | 5.866940376 | 0.621472955 | 3.598977403 | 4.816099989 |
| TCGA-BR-6710 | 0.319759537 | 6.786541237 | 0.144988457 | 3.166200137 | 2.334277727 |
| TCGA-BR-6801 | 0.367283851 | 7.731238942 | 0.785601299 | 4.676700194 | 4.983146478 |
| TCGA-BR-6802 | 0.313603907 | 4.228446219 | 0.013474227 | 4.109047811 | 4.250465916 |
| TCGA-BR-6803 | 0.3905016 | 10.62859108 | -0.132173595 | 3.548445698 | 4.059025564 |
| TCGA-BR-6852 | 0.36332085 | 3.654725018 | 0.44548077 | 3.547345749 | 3.770247795 |
| TCGA-BR-7196 | 0.452569425 | 8.336659283 | 0.029238485 | 4.354404952 | 5.357719011 |
| TCGA-BR-7197 | 0.349976433 | 7.804758653 | -0.742638612 | 4.466571544 | 4.110088768 |
| TCGA-BR-7704 | 0.367935719 | 4.263831169 | -1.258670049 | 4.02654584 | 3.652867715 |
| TCGA-BR-7707 | 0.20819274 | 3.07307431 | 0.251281906 | 3.927451125 | 3.465177024 |
| TCGA-BR-7715 | 0.42496335 | 7.137924365 | -1.082471414 | 4.31162272 | 3.655160439 |
| TCGA-BR-7717 | 0.440902614 | 6.851465504 | -0.297021126 | 4.2075423 | 7.204694459 |
| TCGA-BR-7722 | 0.360477995 | 5.328176166 | 0.441666154 | 4.280576117 | 3.818976528 |
| TCGA-BR-7723 | 0.465485568 | 5.809894449 | 0.144912487 | 4.070714006 | 5.791440012 |
| TCGA-BR-7851 | 0.378470095 | 6.921986413 | 1.350588867 | 4.494500718 | 4.167670002 |
| TCGA-BR-7901 | 0.42039648 | 7.82121922 | 1.542360145 | 4.907785688 | 5.947430158 |
| TCGA-BR-7957 | 0.49800536 | 9.006200193 | -0.366080236 | 3.398533657 | 6.372455731 |
| TCGA-BR-7958 | 0.315129696 | 4.860077037 | -1.229313787 | 3.953529292 | 2.329122562 |
| TCGA-BR-7959 | 0.566601465 | 8.020180978 | -0.563287766 | 4.127665942 | 6.216948947 |
| TCGA-BR-8058 | 0.330850413 | 5.211513282 | 1.02439405 | 3.366482721 | 4.258105184 |
| TCGA-BR-8059 | 0.211477537 | 8.407116332 | 0.449799438 | 4.763292947 | 6.732632341 |
| TCGA-BR-8060 | 0.39935433 | 6.49108308 | -0.798958957 | 4.24983138 | 6.570110731 |
| TCGA-BR-8080 | 0.557683804 | 8.073935445 | 0.883914291 | 4.18203011 | 6.034618416 |
| TCGA-BR-8081 | 0.428006638 | 5.098928041 | -0.13285391 | 4.018733052 | 4.868377396 |
| TCGA-BR-8284 | 0.373899079 | 7.331968482 | -0.923680208 | 4.152609537 | 5.043521566 |
| TCGA-BR-8286 | 0.308595387 | 5.228763048 | 0.967720592 | 3.461034458 | 4.526849783 |
| TCGA-BR-8289 | 0.470972637 | 8.446492421 | -0.860688975 | 3.989363835 | 5.427534626 |
| TCGA-BR-8291 | 0.444388828 | 5.730961342 | -0.153564582 | 4.136285508 | 6.184130147 |
| TCGA-BR-8295 | 0.402903659 | 7.690558224 | -0.265556791 | 3.167865572 | 5.304078564 |
| TCGA-BR-8296 | 0.415739384 | 5.461590147 | -1.14902351 | 3.418364387 | 4.986816695 |
| TCGA-BR-8297 | 0.366672754 | 11.15107846 | 0.777151927 | 4.197311165 | 5.648390411 |
| TCGA-BR-8361 | 0.255588639 | 4.103956547 | 0.637375223 | 3.802669513 | 3.192970203 |
| TCGA-BR-8364 | 0.453272685 | 10.27927552 | -0.693116126 | 2.996322805 | 5.467290408 |
| TCGA-BR-8365 | 0.387951178 | 11.50961552 | -0.019733399 | 4.298321927 | 4.695111461 |
| TCGA-BR-8367 | 0.332617237 | 8.020715956 | -0.000340344 | 3.839681381 | 5.44014851 |
| TCGA-BR-8368 | 0.25496494 | 3.686037148 | -0.883490729 | 4.61142595 | 5.349036004 |
| TCGA-BR-8369 | 0.39203478 | 9.374126371 | -0.912684995 | 4.360820152 | 6.767982407 |
| TCGA-BR-8371 | 0.40789392 | 11.38460296 | 0.313252987 | 4.564863128 | 4.679526849 |
| TCGA-BR-8372 | 0.306776468 | 5.353776495 | -0.887586186 | 4.016850693 | 5.410068659 |
| TCGA-BR-8373 | 0.309781616 | 6.458650219 | -0.457108171 | 3.706244709 | 4.47726088 |
| TCGA-BR-8381 | 0.319347892 | 4.198232252 | 1.771475632 | 3.67972767 | 4.734817358 |
| TCGA-BR-8382 | 0.254054715 | 4.542777101 | -0.101504199 | 3.809278256 | 2.566389816 |
| TCGA-BR-8384 | 0.379347555 | 10.52970821 | 0.100198015 | 4.67618797 | 3.60298354 |
| TCGA-BR-8483 | 0.389497932 | 6.205001601 | -1.031302184 | 4.10215022 | 3.696240436 |
| TCGA-BR-8484 | 0.337824262 | 4.439886693 | 1.243047309 | 3.980912901 | 4.289911093 |
| TCGA-BR-8485 | 0.407412709 | 4.385381777 | -0.343902662 | 3.839639755 | 4.235476024 |
| TCGA-BR-8487 | 0.256585158 | 2.998193659 | 0.282303546 | 3.393762999 | 4.095106048 |
| TCGA-BR-8588 | 0.364838639 | 7.084383879 | 0.558443832 | 3.275246844 | 4.729613103 |
| TCGA-BR-8589 | 0.453060779 | 1.40701447 | 0.464993179 | 3.631444896 | 5.068887432 |
| TCGA-BR-8590 | 0.375294339 | 7.882835212 | 1.868103679 | 4.166786848 | 6.91775536 |
| TCGA-BR-8591 | 0.342127315 | 6.515204867 | -0.244785992 | 4.911830687 | 4.627957633 |
| TCGA-BR-8592 | 0.460740629 | 10.97407403 | -0.477580614 | 3.899627954 | 6.287062021 |
| TCGA-BR-8676 | 0.322914526 | 4.19176698 | 0.225280626 | 4.160750113 | 3.673795017 |
| TCGA-BR-8677 | 0.493010197 | 4.616772374 | 0.285110012 | 4.12011887 | 3.954560198 |
| TCGA-BR-8678 | 0.37210138 | 4.233559879 | -0.450633855 | 4.608260611 | 4.731109251 |
| TCGA-BR-8680 | 0.358501086 | 4.196496362 | 1.815209948 | 3.100645321 | 5.254184192 |
| TCGA-BR-8682 | 0.253700236 | 8.748342735 | -1.648775712 | 3.707921179 | 3.528330987 |
| TCGA-BR-8683 | 0.449056786 | 7.679345555 | -0.364272039 | 4.648727705 | 4.683361815 |
| TCGA-BR-8686 | 0.31622555 | 6.866381527 | -0.772975622 | 4.422330942 | 4.459069069 |
| TCGA-BR-8687 | 0.356721908 | 6.21025601 | -0.082292238 | 3.815620207 | 5.780974755 |
| TCGA-BR-8690 | 0.37369392 | 5.350527247 | 0.11729332 | 2.879967783 | 3.578895626 |
| TCGA-BR-A44T | 0.390376099 | 11.20533303 | -0.303611544 | 3.386450561 | 3.964542119 |
| TCGA-BR-A44U | 0.30536521 | 4.263639103 | 0.657828595 | 4.378518174 | 4.908140425 |
| TCGA-BR-A4CS | 0.360037592 | 4.533478327 | 1.560247107 | 5.136622076 | 7.500686072 |
| TCGA-BR-A4IV | 0.546522143 | 12.02501681 | -0.133836076 | 3.890979942 | 5.792243812 |
| TCGA-BR-A4J5 | 0.353739783 | 9.401023043 | 0.223345184 | 4.191949033 | 6.655826159 |
| TCGA-BR-A4J7 | 0.322503302 | 10.11561627 | -0.276608196 | 3.759861257 | 4.822432471 |
| TCGA-BR-A4J8 | 0.307299972 | 7.530302654 | 0.820835308 | 4.199051004 | 5.346277776 |
| TCGA-BR-A4PF | 0.279502262 | 2.823372171 | 0.186216304 | 4.255932393 | 5.235452371 |
| TCGA-BR-A4QL | 0.216961175 | 4.140564343 | 0.324693457 | 4.046837969 | 4.243664751 |
| TCGA-CD-5798 | 0.408357716 | 10.48620402 | 0.071244744 | 5.356334584 | 5.043192747 |
| TCGA-CD-5799 | 0.291002153 | 4.929300567 | -0.390303402 | 3.332120556 | 6.124621837 |
| TCGA-CD-5800 | 0.432349266 | 8.713765642 | 0.948956442 | 2.729608934 | 6.763339256 |
| TCGA-CD-5801 | 0.379227405 | 2.862902869 | -1.313348306 | 4.643752245 | 2.572917596 |
| TCGA-CD-5803 | 0.332447178 | 8.141723064 | -0.190382572 | 4.446877934 | 3.585147154 |
| TCGA-CD-8524 | 0.462216323 | 7.308687568 | -0.016672381 | 3.541636111 | 7.504622646 |
| TCGA-CD-8525 | 0.469231052 | 5.680164367 | -0.159727376 | 4.443751024 | 4.470642004 |
| TCGA-CD-8526 | 0.383389347 | 6.481454737 | 0.32483255 | 4.028898749 | 4.361345644 |
| TCGA-CD-8527 | 0.43654581 | 8.398303994 | 0.520671692 | 4.278462097 | 5.058463087 |
| TCGA-CD-8528 | 0.22022072 | 3.709511485 | -0.59196452 | 2.753638076 | 4.389642757 |
| TCGA-CD-8529 | 0.455635357 | 7.877862269 | -0.641977574 | 4.318256386 | 5.536735214 |
| TCGA-CD-8530 | 0.417130986 | 8.20529251 | 0.554869682 | 3.780303453 | 4.366399887 |
| TCGA-CD-8531 | 0.299473944 | 4.576955592 | 1.385917306 | 3.996603592 | 6.587770643 |
| TCGA-CD-8532 | 0.370276585 | 5.778603608 | 0.029226101 | 3.937256898 | 4.961246001 |
| TCGA-CD-8533 | 0.449275501 | 4.07150203 | 0.187718779 | 3.514197093 | 6.675731249 |
| TCGA-CD-8534 | 0.343591992 | 7.348320536 | -1.500470136 | 3.253874681 | 3.762757 |
| TCGA-CD-8535 | 0.424592181 | 4.120854169 | 1.2364009 | 4.094466542 | 6.265437383 |
| TCGA-CD-A486 | 0.565109614 | 9.52114641 | -0.971292269 | 4.234059977 | 5.824356903 |
| TCGA-CD-A487 | 0.356937797 | 7.734199013 | 0.605233211 | 3.718810718 | 3.756083791 |
| TCGA-CD-A489 | 0.414700677 | 11.67598768 | -0.471034507 | 4.40907296 | 6.916500927 |
| TCGA-CD-A48A | 0.435067782 | 7.827629061 | -0.924074356 | 3.804561979 | 4.888791794 |
| TCGA-CD-A48C | 0.486079929 | 5.004543522 | -0.212747712 | 4.441573049 | 8.003903733 |
| TCGA-CD-A4MG | 0.305993288 | 4.32889724 | 0.898080934 | 4.813959424 | 5.292249997 |
| TCGA-CD-A4MH | 0.386125726 | 5.02236646 | -0.131443322 | 4.630589676 | 5.455009238 |
| TCGA-CG-4301 | 0.460050438 | 5.566996646 | 0.563513041 | 4.327203555 | 5.226276202 |
| TCGA-CG-4305 | 0.377204513 | 4.484788974 | -0.282071978 | 4.569996356 | 6.268457775 |
| TCGA-CG-4436 | 0.318488906 | 3.522152727 | 0.256186918 | 3.921556272 | 5.416038635 |
| TCGA-CG-4437 | 0.318635509 | 4.469545771 | -1.093794645 | 4.740517737 | 3.713097997 |
| TCGA-CG-4438 | 0.402100155 | 5.041837699 | 2.167873072 | 3.765014403 | 4.387198156 |
| TCGA-CG-4440 | 0.516193682 | 4.710478128 | -0.687908141 | 3.883211958 | 6.664764401 |
| TCGA-CG-4441 | 0.24162098 | 3.935933655 | 0.553965493 | 4.896287467 | 4.737407689 |
| TCGA-CG-4443 | 0.292546852 | 4.811101682 | 0.023276485 | 4.525117742 | 1.923380418 |
| TCGA-CG-4444 | 0.283627963 | 4.290174086 | 0.00367799 | 4.523768453 | 4.046570006 |
| TCGA-CG-4460 | 0.36028794 | 6.458946096 | 0.99996789 | 4.041659094 | 5.999693392 |
| TCGA-CG-4465 | 0.335841526 | 2.978415513 | -0.301645689 | 3.90952684 | 5.254984172 |
| TCGA-CG-4466 | 0.320459429 | 3.329317043 | -0.732821068 | 4.501985999 | 3.610289521 |
| TCGA-CG-4469 | 0.2964822 | 3.016805297 | -0.104316919 | 4.390888332 | 4.934526187 |
| TCGA-CG-4475 | 0.360006409 | 7.432803135 | 0.163113079 | 4.967126456 | 6.171455841 |
| TCGA-CG-4477 | 0.40021717 | 4.467942576 | 0.460338531 | 3.539081592 | 4.285205174 |
| TCGA-CG-5717 | 0.380946453 | 4.990011077 | -1.276420825 | 3.966273269 | 4.547943119 |
| TCGA-CG-5718 | 0.538376821 | 4.079487716 | 0.426722926 | 3.985715389 | 4.791858395 |
| TCGA-CG-5719 | 0.367364387 | 7.54444625 | -1.212067613 | 3.507429536 | 4.898653767 |
| TCGA-CG-5720 | 0.283012469 | 4.080135448 | -1.074133583 | 4.52166945 | 4.790073164 |
| TCGA-CG-5721 | 0.40296715 | 4.570162909 | 1.039070458 | 3.494813617 | 4.001355057 |
| TCGA-CG-5722 | 0.355593035 | 4.001486999 | -0.535363251 | 3.985933177 | 8.006007375 |
| TCGA-CG-5723 | 0.390436449 | 4.938407958 | 0.44304003 | 5.154711368 | 4.743512945 |
| TCGA-CG-5724 | 0.477858783 | 4.80613874 | -1.363814005 | 3.978535158 | 7.853471031 |
| TCGA-CG-5725 | 0.328935915 | 8.502839987 | 1.04317099 | 4.776930245 | 5.648650993 |
| TCGA-CG-5726 | 0.185560408 | 3.893600461 | -0.523889313 | 4.432125306 | 5.478566306 |
| TCGA-CG-5732 | 0.565046315 | 4.417263038 | 0.912170537 | 4.252447472 | 5.210401566 |
| TCGA-CG-5734 | 0.330575285 | 3.78603099 | 1.946831259 | 4.229662356 | 3.899369373 |
| TCGA-D7-5577 | 0.387937437 | 3.294136076 | -0.002491492 | 3.154096715 | 6.070891799 |
| TCGA-D7-5578 | 0.383203688 | 5.097864856 | -0.384236808 | 4.812883403 | 5.622702091 |
| TCGA-D7-6519 | 0.403059727 | 4.687788693 | 0.989741962 | 3.733453869 | 7.625578366 |
| TCGA-D7-6520 | 0.223568778 | 6.773492845 | 0.519692114 | 3.880789499 | 4.304312956 |
| TCGA-D7-6521 | 0.400575355 | 6.02816977 | -0.837624009 | 4.592836779 | 4.961152821 |
| TCGA-D7-6522 | 0.426353296 | 7.043046681 | -0.024765931 | 4.074747738 | 5.077913637 |
| TCGA-D7-6524 | 0.312450056 | 7.049438582 | -0.437395963 | 3.927231241 | 6.844416351 |
| TCGA-D7-6525 | 0.374039265 | 4.741061645 | 1.463890982 | 4.113492072 | 6.781764217 |
| TCGA-D7-6526 | 0.40669459 | 7.746062791 | 0.972380163 | 3.643551867 | 5.509170601 |
| TCGA-D7-6527 | 0.401577765 | 6.459450004 | 0.508020217 | 4.132841277 | 4.569009394 |
| TCGA-D7-6528 | 0.320077842 | 3.760610229 | 0.079422571 | 3.760092571 | 5.821602327 |
| TCGA-D7-6815 | 0.28600829 | 4.33434232 | 0.113541365 | 3.939330497 | 3.33593361 |
| TCGA-D7-6818 | 0.440933574 | 6.651777334 | 0.968679562 | 3.721397269 | 8.164839816 |
| TCGA-D7-6822 | 0.328628422 | 4.972957601 | 0.359265398 | 4.843512767 | 5.647849041 |
| TCGA-D7-8570 | 0.369560581 | 3.931438848 | 0.350262734 | 3.40953666 | 3.66730768 |
| TCGA-D7-8572 | 0.442732013 | 6.87329927 | -0.393865893 | 3.883387767 | 5.674829428 |
| TCGA-D7-8573 | 0.445027973 | 3.682440274 | -0.506546039 | 3.901210778 | 4.431074868 |
| TCGA-D7-8574 | 0.358069531 | 4.109704814 | 0.742023682 | 4.710443054 | 4.075443839 |
| TCGA-D7-8575 | 0.425630221 | 5.218124799 | 0.045067262 | 4.382795603 | 6.02983085 |
| TCGA-D7-8576 | 0.340722295 | 4.35630948 | 0.758419133 | 3.291715688 | 6.967943715 |
| TCGA-D7-8578 | 0.410220654 | 8.323081813 | -0.596699809 | 4.864501678 | 7.536457182 |
| TCGA-D7-8579 | 0.394960148 | 9.566797404 | 1.154705255 | 3.998406807 | 5.932631802 |
| TCGA-D7-A4YU | 0.363481825 | 6.639573963 | -0.653844823 | 4.679002707 | 3.676799762 |
| TCGA-D7-A4YX | 0.284759132 | 2.381020783 | -1.25308409 | 3.876148576 | 4.609013272 |
| TCGA-D7-A4Z0 | 0.380761571 | 6.950966403 | 1.406289704 | 3.641799309 | 6.384167511 |
| TCGA-D7-A6EV | 0.226240579 | 3.841162044 | 0.026294947 | 3.734677217 | 4.673132553 |
| TCGA-D7-A6EX | 0.251859034 | 8.624688025 | -0.099625729 | 4.193977159 | 3.945388729 |
| TCGA-D7-A6EY | 0.315878375 | 6.557585621 | -1.04349619 | 3.970125679 | 4.379010546 |
| TCGA-D7-A6EZ | 0.288668977 | 3.201861036 | 0.457222079 | 4.296355506 | 3.565898973 |
| TCGA-D7-A6F0 | 0.391335977 | 7.69376882 | 1.115497563 | 4.147978758 | 6.845597155 |
| TCGA-D7-A6F2 | 0.332887005 | 5.17711624 | -0.406557684 | 4.026102625 | 4.253157387 |
| TCGA-D7-A747 | 0.368126251 | 9.617173482 | -0.744311234 | 4.264152073 | 4.059388567 |
| TCGA-D7-A748 | 0.539421379 | 4.313290673 | 1.60416112 | 3.07659384 | 8.007136587 |
| TCGA-D7-A74A | 0.303259846 | 4.322351988 | 0.271202378 | 3.788121204 | 4.984880766 |
| TCGA-EQ-8122 | 0.520687831 | 8.912311706 | -0.372805539 | 4.122596535 | 5.250366592 |
| TCGA-F1-6874 | 0.327692892 | 4.038700425 | 0.211328635 | 4.632589479 | 5.235094102 |
| TCGA-F1-6875 | 0.463903673 | 4.820767972 | -0.652785106 | 4.733310066 | 4.676108619 |
| TCGA-F1-A448 | 0.43086344 | 5.134719191 | -1.203959607 | 4.404699053 | 5.863672906 |
| TCGA-F1-A72C | 0.320211207 | 5.052998547 | -0.531397464 | 4.534630865 | 5.021136216 |
| TCGA-FP-7735 | 0.49688535 | 5.95997964 | -0.061702344 | 5.044451144 | 6.577944674 |
| TCGA-FP-7829 | 0.314579623 | 4.411624887 | 0.747433899 | 4.433875787 | 4.900694965 |
| TCGA-FP-7916 | 0.339018636 | 6.586909567 | 0.595598105 | 4.945933704 | 4.493966101 |
| TCGA-FP-7998 | 0.362408841 | 6.43005936 | -0.17462476 | 4.81622503 | 3.3654032 |
| TCGA-FP-8099 | 0.386770429 | 4.282166911 | 1.011210301 | 4.939161656 | 4.3999059 |
| TCGA-FP-8209 | 0.428165766 | 10.64442299 | 0.056630571 | 3.775821481 | 3.861211741 |
| TCGA-FP-8210 | 0.429870627 | 9.944879001 | 0.700666901 | 3.76229225 | 5.6659678 |
| TCGA-FP-8211 | 0.550959882 | 3.777056523 | -0.174524649 | 4.239091217 | 3.064103726 |
| TCGA-FP-A4BF | 0.501847105 | 4.331361401 | 0.435257883 | 4.511891649 | 6.484205203 |
| TCGA-FP-A9TM | 0.298981374 | 4.376015693 | 0.74693254 | 4.668997036 | 4.40166282 |
| TCGA-HF-7132 | 0.323136197 | 6.091442246 | -0.575287908 | 1.350433618 | 4.617214882 |
| TCGA-HF-7133 | 0.499589173 | 5.335085106 | -0.563250926 | 0.622571441 | 5.673563887 |
| TCGA-HF-7134 | 0.305617595 | 4.14132699 | 1.296140846 | 1.179631708 | 5.411924207 |
| TCGA-HF-A5NB | 0.281796986 | 3.497921826 | -0.016323219 | 3.924587265 | 4.495999152 |
| TCGA-HJ-7597 | 0.337829277 | 5.331656814 | 1.010922968 | 4.059082259 | 4.795303636 |
| TCGA-HU-8238 | 0.360177169 | 3.93194077 | -0.217899714 | 3.884547101 | 6.475036053 |
| TCGA-HU-8244 | 0.211732192 | 2.517034765 | 0.11320703 | 4.222289587 | 5.330115621 |
| TCGA-HU-8249 | 0.313044163 | 3.735091063 | 1.290614775 | 4.627409228 | 5.029518595 |
| TCGA-HU-8602 | 0.315127206 | 2.082510207 | -0.78166728 | 3.40967798 | 4.027272841 |
| TCGA-HU-8604 | 0.275624996 | 4.248008269 | 0.1261939 | 4.530968421 | 4.335853913 |
| TCGA-HU-8608 | 0.470180178 | 2.597783791 | 1.349771335 | 4.134545503 | 2.338818252 |
| TCGA-HU-A4G2 | 0.347421374 | 4.584653577 | 0.228985731 | 3.333514636 | 6.152322403 |
| TCGA-HU-A4G3 | 0.366936998 | 5.199595392 | -1.7114711 | 3.634116395 | 5.974882811 |
| TCGA-HU-A4G8 | 0.29936923 | 3.396808066 | -0.188674113 | 3.757021969 | 2.969681073 |
| TCGA-HU-A4G9 | 0.161371814 | 2.422142415 | 0.830827224 | 3.556747597 | 3.914984345 |
| TCGA-HU-A4GD | 0.393599994 | 5.041309418 | 0.704912828 | 3.591406649 | 6.137903536 |
| TCGA-HU-A4GF | 0.397009873 | 5.359582251 | 0.377052818 | 4.289662574 | 4.595224281 |
| TCGA-HU-A4GH | 0.330387719 | 6.878072809 | 1.443731544 | 4.522684469 | 4.855028728 |
| TCGA-HU-A4GJ | 0.41581804 | 5.736925216 | -0.173192457 | 3.384058995 | 3.714360873 |
| TCGA-HU-A4GP | 0.294004977 | 5.17033264 | 0.344029972 | 3.131783273 | 5.297045225 |
| TCGA-HU-A4GT | 0.241771921 | 3.770050175 | 0.287801201 | 3.845406152 | 4.428821325 |
| TCGA-HU-A4GU | 0.314997607 | 2.703490266 | -1.314409387 | 4.19874551 | 3.477495637 |
| TCGA-HU-A4GX | 0.263545906 | 4.813271427 | 1.332252172 | 3.633117397 | 4.341712151 |
| TCGA-HU-A4H0 | 0.352507104 | 2.965360239 | 0.51380831 | 4.509720396 | 4.426858771 |
| TCGA-HU-A4H2 | 0.264105973 | 5.015160846 | -0.046634435 | 3.167786734 | 4.082796181 |
| TCGA-HU-A4H3 | 0.397116303 | 4.440076304 | 0.382830609 | 3.272907309 | 4.809100015 |
| TCGA-HU-A4H4 | 0.314677814 | 3.772184026 | -1.327362017 | 2.972325117 | 3.118171346 |
| TCGA-HU-A4H5 | 0.301509585 | 4.424186177 | 1.001095613 | 4.273978389 | 5.144249099 |
| TCGA-HU-A4H6 | 0.339512438 | 5.765737879 | 0.518040128 | 3.953730749 | 4.099327512 |
| TCGA-HU-A4H8 | 0.195828301 | 3.427983879 | 0.929337705 | 4.741372716 | 4.577960254 |
| TCGA-HU-A4HB | 0.528536429 | 4.959109591 | -0.613857115 | 4.179031349 | 6.249059638 |
| TCGA-HU-A4HD | 0.452846088 | 7.924198872 | 0.190927555 | 4.674869179 | 5.71078959 |
| TCGA-IN-7806 | 0.278206029 | 6.264790786 | -0.396254202 | 3.483289313 | 5.407981131 |
| TCGA-IN-7808 | 0.425690856 | 3.631584296 | -1.440363357 | 3.938452644 | 4.442782443 |
| TCGA-IN-8462 | 0.437363481 | 6.967859155 | -0.183472803 | 5.026098055 | 5.17948798 |
| TCGA-IN-8663 | 0.395445748 | 4.906476259 | 0.597965001 | 4.299933882 | 6.90411597 |
| TCGA-IN-A6RI | 0.310051427 | 5.760907896 | -0.188293803 | 3.057003941 | 5.312210103 |
| TCGA-IN-A6RJ | 0.216518236 | 3.538542453 | -0.533880199 | 4.227085263 | 3.612835237 |
| TCGA-IN-A6RL | 0.333543835 | 5.816563714 | 0.640515274 | 5.219830792 | 6.426337368 |
| TCGA-IN-A6RN | 0.271070933 | 5.991564238 | 0.556658562 | 3.732274243 | 5.25280298 |
| TCGA-IN-A6RR | 0.341005574 | 4.049077241 | -0.648735934 | 5.091050397 | 6.463499144 |
| TCGA-IN-A6RS | 0.352580225 | 3.026833425 | -0.087389117 | 4.641046615 | 5.865066836 |
| TCGA-IN-A7NR | 0.284856161 | 4.408281598 | 0.570382421 | 3.744430604 | 6.736496115 |
| TCGA-IN-A7NT | 0.230407672 | 6.733811583 | -0.62821386 | 4.167053149 | 4.844886464 |
| TCGA-IN-A7NU | 0.296356066 | 4.166899793 | 0.336364306 | 4.570630467 | 6.338457449 |
| TCGA-IN-AB1V | 0.302954585 | 6.048272778 | 0.886295706 | 4.27230396 | 4.950218087 |
| TCGA-IN-AB1X | 0.338733232 | 3.434896547 | -0.465914485 | 4.191137806 | 4.549277555 |
| TCGA-IP-7968 | 0.431925333 | 5.054726302 | -0.665758172 | 4.672217196 | 5.201012417 |
| TCGA-KB-A6F7 | 0.51249879 | 7.099733958 | -0.161250066 | 3.066493024 | 5.540170514 |
| TCGA-KB-A93G | 0.490407233 | 6.157898861 | -1.14082912 | 4.486133281 | 5.425320033 |
| TCGA-KB-A93H | 0.335371929 | 5.062505644 | -0.267274411 | 4.196012756 | 5.776937814 |
| TCGA-KB-A93J | 0.409683104 | 3.109356184 | -0.699992041 | 4.99815974 | 4.365051228 |
| TCGA-MX-A5UG | 0.389084721 | 7.396947858 | 0.509022157 | 4.950827024 | 6.87718522 |
| TCGA-MX-A5UJ | 0.449266361 | 8.067416792 | 0.017731536 | 4.813657749 | 6.256322363 |
| TCGA-MX-A663 | 0.580071558 | 9.585043388 | 0.199486338 | 4.571392902 | 8.414202025 |
| TCGA-MX-A666 | 0.413784466 | 6.350965577 | -0.069604662 | 3.893738406 | 5.97850585 |
| TCGA-R5-A7O7 | 0.350780724 | 4.021550868 | 0.314384411 | 3.482403061 | 3.010841816 |
| TCGA-R5-A7ZE | 0.370461634 | 5.030428755 | 0.389368532 | 3.940228045 | 4.790218354 |
| TCGA-R5-A7ZF | 0.296619419 | 2.079432789 | 0.309552898 | 3.850317469 | 4.417404279 |
| TCGA-R5-A7ZI | 0.22609879 | 4.027297112 | -0.331404048 | 2.465430178 | 3.419515416 |
| TCGA-R5-A7ZR | 0.332560945 | 2.793916588 | 0.82389411 | 3.800572705 | 4.643530274 |
| TCGA-R5-A805 | 0.424494309 | 5.055155447 | -0.608261839 | 4.483355381 | 5.301966383 |
| TCGA-RD-A7BS | 0.380584143 | 4.797805231 | -0.239354185 | 3.502772809 | 5.23299055 |
| TCGA-RD-A7BT | 0.296904041 | 4.43072923 | -0.416169354 | 4.248415701 | 3.475308211 |
| TCGA-RD-A7BW | 0.381322309 | 10.24888808 | -0.200555661 | 4.427515279 | 5.14692291 |
| TCGA-RD-A7C1 | 0.285289984 | 3.59416639 | 0.849703928 | 4.9235223 | 4.72298799 |
| TCGA-RD-A8MV | 0.416610027 | 3.710025269 | -0.152978322 | 3.673084766 | 5.112784652 |
| TCGA-RD-A8MW | 0.533121247 | 7.727553927 | -0.123855639 | 4.73075825 | 4.958815187 |
| TCGA-RD-A8N0 | 0.399023351 | 7.139516538 | -0.40349662 | 3.229080611 | 3.899081644 |
| TCGA-RD-A8N1 | 0.374958427 | 4.606258289 | 1.122909057 | 4.385980714 | 3.557585758 |
| TCGA-RD-A8N2 | 0.305474872 | 11.62985302 | -0.095861997 | 4.066268007 | 3.686980842 |
| TCGA-RD-A8N4 | 0.418364323 | 12.64975454 | -0.430051042 | 3.879920452 | 3.250663647 |
| TCGA-RD-A8N5 | 0.347931385 | 4.831794583 | 0.597235806 | 5.034146436 | 5.713829567 |
| TCGA-RD-A8N6 | 0.506625398 | 9.511990816 | -0.376831236 | 4.603608082 | 7.647078838 |
| TCGA-RD-A8N9 | 0.421546953 | 7.174123145 | 0.579434467 | 3.864687574 | 5.49294948 |
| TCGA-RD-A8NB | 0.34709228 | 7.963833295 | 0.689103729 | 4.486286906 | 5.12331355 |
| TCGA-SW-A7EA | 0.294084713 | 2.868628809 | -0.397642601 | 3.423534524 | 4.207492063 |
| TCGA-SW-A7EB | 0.390171379 | 5.346253453 | -1.53756941 | 3.196858447 | 5.165694348 |
| TCGA-VQ-A8DT | 0.311866203 | 4.778164665 | -0.022658532 | 3.205394562 | 6.173151182 |
| TCGA-VQ-A8DU | 0.366695772 | 5.150276182 | -0.040814751 | 3.978674962 | 4.634000655 |
| TCGA-VQ-A8DV | 0.582390519 | 6.885777763 | 0.466443306 | 3.853240812 | 10.08136759 |
| TCGA-VQ-A8DZ | 0.429136191 | 7.224639964 | -0.752649467 | 4.357454476 | 7.169365365 |
| TCGA-VQ-A8E0 | 0.53459593 | 7.610651674 | -0.372018312 | 4.616346308 | 5.105183104 |
| TCGA-VQ-A8E2 | 0.448102493 | 7.63014158 | -0.889166934 | 3.793032997 | 5.890574563 |
| TCGA-VQ-A8E3 | 0.375373335 | 3.193635163 | 0.858396812 | 4.99167069 | 5.271731525 |
| TCGA-VQ-A8E7 | 0.375357438 | 4.1043167 | 0.0603317 | 3.880737932 | 5.286456364 |
| TCGA-VQ-A8P2 | 0.239143678 | 2.98688829 | -0.063207777 | 4.374718847 | 5.456555469 |
| TCGA-VQ-A8P3 | 0.422861836 | 5.290313092 | 0.913224578 | 4.429565786 | 3.866198747 |
| TCGA-VQ-A8P5 | 0.396668996 | 4.784897497 | 0.399145263 | 4.214313997 | 4.532122339 |
| TCGA-VQ-A8P8 | 0.323093031 | 6.754915898 | 0.667473587 | 4.290258958 | 4.490773136 |
| TCGA-VQ-A8PB | 0.255540313 | 3.687891072 | 0.298627034 | 3.814412655 | 4.836008088 |
| TCGA-VQ-A8PC | 0.35044344 | 5.912994635 | 1.052014468 | 4.181788674 | 5.575741142 |
| TCGA-VQ-A8PD | 0.385894061 | 6.129370695 | 0.7549131 | 4.38355172 | 6.262301823 |
| TCGA-VQ-A8PE | 0.372756534 | 5.462202535 | -0.020817017 | 4.623140357 | 4.168538978 |
| TCGA-VQ-A8PF | 0.387749416 | 3.595428033 | 1.208095033 | 4.750399074 | 4.330740742 |
| TCGA-VQ-A8PH | 0.430090007 | 4.453905864 | -0.795853122 | 4.049787772 | 4.940210074 |
| TCGA-VQ-A8PJ | 0.440658202 | 6.298347856 | 0.513184353 | 3.653283157 | 7.562664152 |
| TCGA-VQ-A8PK | 0.450080989 | 6.251226028 | -1.464827236 | 3.758733644 | 4.640894505 |
| TCGA-VQ-A8PM | 0.433545043 | 5.87795517 | 0.533117416 | 3.966477785 | 6.485847115 |
| TCGA-VQ-A8PO | 0.281027805 | 2.941248551 | 0.032564419 | 4.645348392 | 3.783124808 |
| TCGA-VQ-A8PP | 0.393442645 | 4.764323018 | 0.389348578 | 4.866421703 | 5.244389733 |
| TCGA-VQ-A8PQ | 0.426143007 | 3.915453658 | -0.411272808 | 3.125628177 | 4.478183547 |
| TCGA-VQ-A8PU | 0.335834071 | 4.331882041 | -0.193422105 | 3.897630191 | 2.849761884 |
| TCGA-VQ-A8PX | 0.364014115 | 3.028577163 | 1.635835586 | 3.280723388 | 4.118311907 |
| TCGA-VQ-A91A | 0.469738607 | 5.679319366 | -0.032245674 | 4.537257944 | 5.915939277 |
| TCGA-VQ-A91D | 0.356788804 | 3.495213948 | -0.529439019 | 4.594652897 | 5.288479055 |
| TCGA-VQ-A91E | 0.365935864 | 4.098537136 | 0.098088584 | 3.676065326 | 5.341630247 |
| TCGA-VQ-A91K | 0.234273406 | 7.037800065 | 0.800416001 | 4.431703963 | 4.806286452 |
| TCGA-VQ-A91N | 0.323811495 | 4.796731108 | 0.201789315 | 3.244179592 | 5.496189471 |
| TCGA-VQ-A91Q | 0.452712996 | 5.916687243 | 0.375377249 | 4.23250828 | 4.581551548 |
| TCGA-VQ-A91S | 0.353945575 | 3.622750038 | 1.022344833 | 3.828048441 | 3.433933914 |
| TCGA-VQ-A91U | 0.248060302 | 3.251198165 | -0.288008313 | 4.61594077 | 2.991468907 |
| TCGA-VQ-A91V | 0.284542885 | 5.892167483 | 0.397299817 | 3.914776573 | 6.832733958 |
| TCGA-VQ-A91X | 0.329075278 | 3.177419794 | -1.022652575 | 4.471080453 | 4.499424734 |
| TCGA-VQ-A91Y | 0.487773541 | 9.394600443 | 0.318634641 | 4.610428829 | 5.686935166 |
| TCGA-VQ-A91Z | 0.387096752 | 5.169845743 | -0.137551316 | 4.02226016 | 3.442302318 |
| TCGA-VQ-A922 | 0.445757965 | 6.524289036 | 0.718841518 | 4.38592925 | 6.501007997 |
| TCGA-VQ-A924 | 0.234958023 | 5.560175125 | -0.130065303 | 4.400502003 | 3.820882555 |
| TCGA-VQ-A925 | 0.46556456 | 7.062028272 | 0.822339512 | 4.27457033 | 6.238604173 |
| TCGA-VQ-A927 | 0.320885945 | 5.375679336 | -0.395436019 | 5.024478176 | 5.224552914 |
| TCGA-VQ-A928 | 0.505543469 | 8.076801347 | 0.646680486 | 3.409637564 | 5.493672738 |
| TCGA-VQ-A92D | 0.279430921 | 4.555108097 | -0.287683104 | 4.180072994 | 6.002644777 |
| TCGA-VQ-A94O | 0.528577987 | 3.877065723 | -0.090316271 | 4.839314079 | 6.039668299 |
| TCGA-VQ-A94R | 0.291329754 | 5.718674926 | 0.698634572 | 4.213961218 | 4.637501801 |
| TCGA-VQ-A94T | 0.423463862 | 4.60746702 | 1.133978279 | 4.775505521 | 5.279087096 |
| TCGA-VQ-A94U | 0.297232223 | 6.910565217 | -0.012683513 | 4.574271673 | 5.804246217 |
| TCGA-VQ-AA64 | 0.451412885 | 6.289918026 | -0.370412331 | 4.50555428 | 4.365742965 |
| TCGA-VQ-AA68 | 0.407055457 | 3.999493941 | 0.402661347 | 3.02251364 | 3.45541613 |
| TCGA-VQ-AA69 | 0.381965082 | 2.477268212 | -0.106348068 | 3.003733912 | 4.039101727 |
| TCGA-VQ-AA6A | 0.373348553 | 4.398162241 | -0.542465159 | 3.555837645 | 4.81929244 |
| TCGA-VQ-AA6D | 0.474739985 | 5.461235142 | -0.106244117 | 2.689184818 | 4.681476991 |
| TCGA-VQ-AA6F | 0.453062417 | 4.957808591 | -0.868130581 | 3.729177269 | 4.113647682 |
| TCGA-VQ-AA6G | 0.409899959 | 5.083553754 | 0.716134152 | 4.235484893 | 5.687920405 |
| TCGA-VQ-AA6J | 0.372801538 | 6.237140818 | 1.041281547 | 4.807843284 | 3.542348416 |
| TCGA-VQ-AA6K | 0.468301199 | 4.71527007 | 0.408903461 | 4.202845059 | 3.756331242 |
| TCGA-ZA-A8F6 | 0.332769281 | 9.500101065 | -0.304741145 | 4.702128745 | 6.02086428 |
